# Seasonal bromate formation in the Arctic snowpack: Implications for the bromine biogeochemical cycle

**DOI:** 10.1126/sciadv.aea3286

**Published:** 2026-04-17

**Authors:** Stefano Frassati, Elena Barbaro, Giulio Cozzi, Clara Turetta, Federico Scoto, Claudia Rossetti, Marco Roman, Matteo Feltracco, Kitae Kim, François Burgay, Alfonso Saiz-Lopez, Joseph S. Francisco, Ward Van Pelt, Feiyue Wang, David Cappelletti, Sofia Lerda, Giovanni Bistoni, Filippo De Angelis, Carlo Barbante, Andrea Gambaro, Andrea Spolaor

**Affiliations:** ^1^ Department of Environmental Sciences, Informatics and Statistics, Ca’ Foscari University of Venice, Venice Mestre, Italy.; ^2^ Institute of Polar Sciences–National Research Council of Italy (CNR-ISP), Venice Mestre, Italy.; ^3^ Korea Polar Research Institute (KOPRI), Incheon 21990, Republic of Korea.; ^4^ Department of Environmental Sciences, University of Basel, 4001 Basel, Switzerland.; ^5^ Department of Atmospheric Chemistry and Climate, Institute of Physical Chemistry Blas Cabrera, CSIC, 28006 Madrid, Spain.; ^6^ Department of Earth and Environmental Science and Department of Chemistry, University of Pennsylvania, Philadelphia, PA 19104, USA.; ^7^ Department of Earth Sciences, Uppsala University, Uppsala, Sweden.; ^8^ Centre for Earth Observation Science, and Department of Environment and Geography, University of Manitoba, Winnipeg, Manitoba, Canada.; ^9^ Department of Chemistry, Biology and Biotechnology, University of Perugia, Perugia, 06123, Italy.

## Abstract

Bromine exists in multiple chemical forms in the atmosphere, with bromide (Br^−^) being the predominant species in snow. Here, we detect and track the formation of bromate (BrO_3_^−^) in Arctic snow and propose a mechanism for its production. Our observations reveal relevant BrO_3_^−^ concentrations, reaching up to 5% of total bromine, during springtime. The evidence for the persistence of BrO_3_^−^ in the snowpack and its production as a function of solar radiation suggests a snow-driven photochemical process, with negligible contributions from direct aerosol deposition, and emphasizes the role of the snowpack as a reactive matrix for photooxidation, providing insights into the bromine cycle in the Arctic. Our findings are supported by quantum chemical calculations, which explore both radical and ionic reaction mechanisms and the matrix effect, thereby supporting the role of snowpack in promoting BrO_3_^−^ formation. The presence of BrO_3_^−^ in snowpack represents a reservoir of nonreactive bromine, with potential implications for understanding halogen chemistry in polar environments.

## Teaser

A mechanism for the formation of BrO_3_^−^ in Arctic snow.

## INTRODUCTION

Bromine in polar regions plays an important role in atmospheric processes that influence tropospheric and stratospheric ozone depletion reactions, as well as atmospheric mercury depletion events, with relevant implications for our understanding of the Earth’s radiative budget and energy dynamics (*1*, *2*). The presence of bromine in the snowpack of polar regions is caused by direct emissions and deposition of bromide-containing sea spray aerosols, along with the reemission of gas-phase bromine during springtime from photochemical processes over the sea ice, especially over first-year sea ice (*3*–*5*). In the atmosphere, bromine can be present in several forms, both inorganic (*6*, *7*) (such as Br_2_, BrO, HOBr, BrCl, and BrNO_2_) and organic (such as CH_3_Br), but its main form in the snowpack is bromide (Br^−^) (*8*).

Although bromine activation over first-year sea ice snow is well documented (*3*–*5*), the activation and release of bromine from terrestrial snowpack into the atmosphere remain uncertain. Discordant observations and hypotheses (*9*, *10*) have led to debate regarding the potential storage of bromine in the snowpack (*11*). The photo-stimulated emission of gaseous bromine (Br_2_) from the snow surface into the atmosphere has been observed in Greenland and Alaska (*10*, *12*). However, a specific study of the diurnal remobilization of halogens from the snowpack indicated that there was no substantial variation in bromine levels between day and night, suggesting that there is no emission of brominated species into the atmosphere (*13*).

These discrepancies in experimental data therefore affect the accuracy of models that predict, for example, ozone depletion events. The WRF-Chem model, which does not include a halogen chemistry component, overestimates the amount of ozone compared to observation, especially at Arctic coastal sites in the spring (*14*). In contrast, other models that include halogen reactions tend to underestimate total ozone in the Arctic in the early spring and overestimate the emission of reactive bromine species from snow (*15*, *16*). To minimize these effects, the models assume a pool of nonreactive bromine in the snow (*15*, *17*, *18*). The implementation of halogens in a global model highlights the importance of accounting for halogen chemistry to reproduce springtime surface ozone in polar regions accurately and shows that halogen-induced ozone depletion is exported from the Arctic to mid-latitudes (*19*, *20*).

One of such nonreactive bromine species is bromate (BrO_3_^−^), the ultimate oxidation product of Br^−^ through reactions with oxidants such as O_3_ or •OH (*21*). Numerous studies have documented the presence of oxidant species in snow, including reactive oxygen species (ROS; e.g., O_3_, •OH, •OOH, and H_2_O_2_), some of which are active participants in bromine explosion reactions (*22*–*24*). ROS in the snowpack could be produced through photochemical reactions involving NO_3_^−^ and NO_2_^−^ (at λ_max_ = 320 nm). These reactions, under acidic conditions (pH < 6), can produce O^−^, which rapidly reacts with water to form the •OH (*25*). Alternatively, nitrate photolysis can release atomic oxygen, which may react with dissolved molecular oxygen to form O_3_ (*22*, *26*, *27*). At these same wavelengths, H_2_O_2_ can undergo homolytic photolysis, producing hydroxyl radicals (•OH) (*28*, *29*). Most of these oxidants require ultraviolet (UV) light to be formed in situ in the snowpack. Conversely, ozone can diffuse into the snow from the atmosphere, there being the most effective oxidant in dark conditions. BrO_3_^−^ formation has been well studied in aqueous media, especially in water treatment systems due to its carcinogenic properties (*30*, *31*). The initial reaction step for its formation involves the oxidation of bromide to either a bromine atom or hypobromous acid, HOBr. Both pathways could then lead to further oxidation steps to BrO_3_^−^. However, to the best of our knowledge, no studies have investigated the presence of BrO_3_^−^ in snow.

This study presents the evidence of BrO_3_^−^ in Arctic snowpacks. We experimentally demonstrate that the surface layers of the polar snowpack can create an ideal environment for fostering the oxidation of bromide to BrO_3_^−^ when exposed to sunlight, thereby acting as a nonreactive bromine reservoir that has not yet been considered in current models. We also provide a theoretical framework to understand the oxidation pathways in BrO_3_^−^ formation.

## RESULTS

### Bromate occurrence in the surface snow and seasonal snowpack

**Fig. 1.**
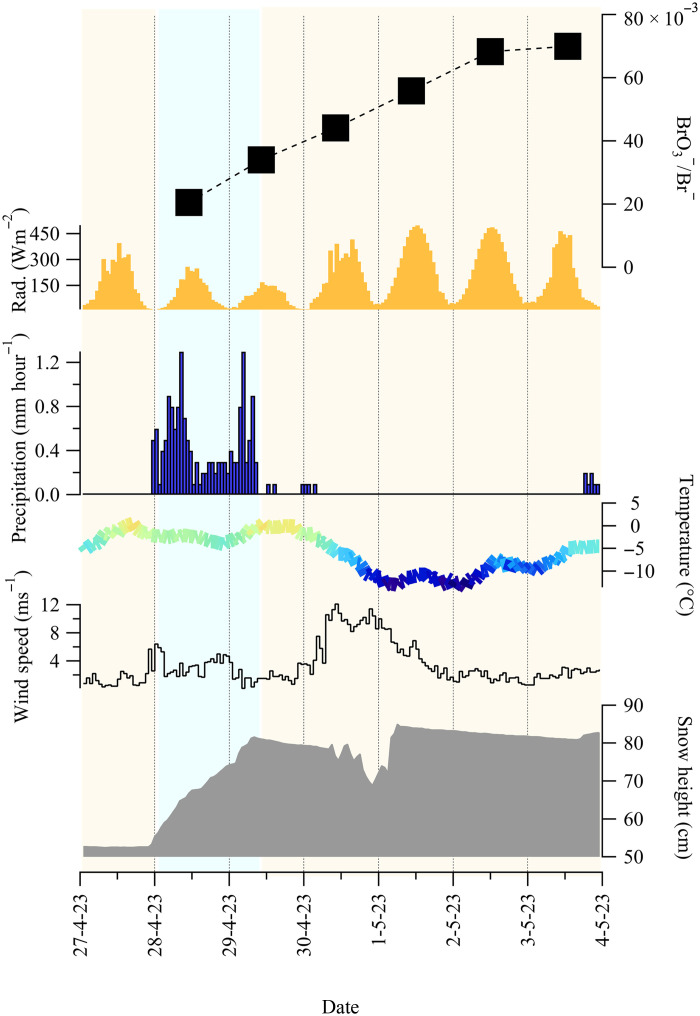
Daily evolution of BrO_3_^−^/Br^−^ ratio levels in surface snow. Trends in BrO_3_^−^ values in relation to meteorological parameters (such as shortwave radiation, precipitation, temperature, and wind speed) and snow height during the period from 28 April to 3 May 2023 at GSRS. Blue band represents the period of precipitation. The meteorological precipitation and snow depth data are shown at hourly frequency (https://seklima.met.no/observations), while the irradiance data are shown at 30-min resolution and come from CCT (*45*). Temperature is represented using a color gradient from blue (colder) to yellow (warmer) to facilitate visual interpretation.

To examine the presence of BrO_3_^−^ in Arctic snowpacks and the mechanism that controls its formation, we designed and conducted specific experiments in the Svalbard archipelago at different sites and with different temporal resolutions. First, to quantify the presence of BrO_3_^−^ in surface snow and evaluate potential in situ production processes, daily snow samples were collected at the Gruvebadet Snow Research Site (GSRS) in Svalbard following a snowfall event, from 28 April to 3 May 2023 (see Materials and Methods). The results revealed a 10-fold increase in BrO_3_^−^ concentration over the 5 days, rising from 0.4 nM on the first day to 4.3 nM on the last day (see fig. S5). Concurrently, bromide (Br^−^) concentration increased from 20 to 63 nM, showing a similar trend to BrO_3_^−^ (fig. S5). To determine whether the increase in BrO_3_^−^ was solely related to a parallel rise in bromide, BrO_3_^−^ concentrations were normalized to Br^−^ concentrations. During the study period, the BrO_3_^−^/Br^−^ ratio increased from 2.05 × 10^−2^ (28 April) to 7.00 × 10^−2^ (3 May), as shown in Fig. 1.

**Fig. 2.**
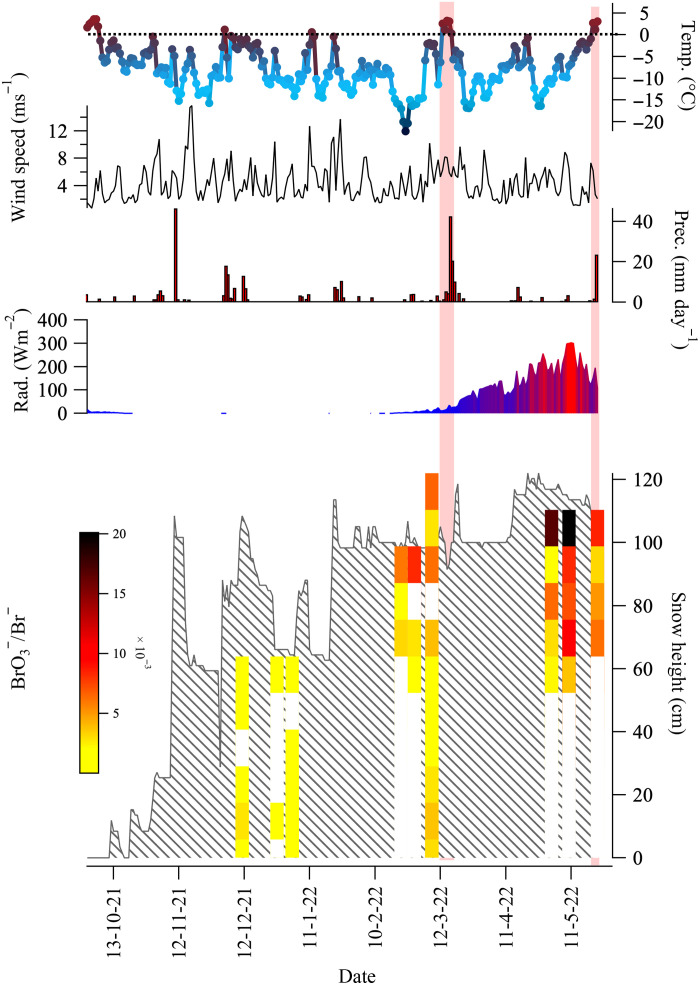
Seasonal evolution of BrO_3_^−^/Br^−^ ratio levels in snow. Time series of bromate in the seasonal snowpack at GSRS in relation to temperature, wind speed, precipitation, and radiation. Samples were collected during the polar night, dawn, and full day. The discrepancy between the height measured during the sampling and the height measured by the automatic measuring device is due to the punctual instrumental measurement, while the sampling area covers an area of 6 m by 10 m with possible surface irregularities and different accumulations of snow. The red band represents the rainfall period. Temperature is represented using a color gradient from blue (colder) to yellow (warmer), and shortwave radiation is shown from blue (lower) to red (higher), to facilitate visual interpretation.

The second experiment was aimed at investigating the effect of varying solar radiation on the potential bromate production within the seasonal snowpack. We analyzed a series of snow pit samples collected at the GSRS through weekly sampling during the 2021–2022 snow season. Three sets of snowpit samples were collected during the polar night, three during the day/night cycle period and three during the polar day, thus with a full exposure of the upper part of the snowpack to sunlight. BrO_3_^−^ concentrations in the snowpack collected during the polar night and in the day-night cycle period were similar, ranging between 2.0 × 10^−2^ to 3.6 × 10^−1^ nM, with a mean value of the BrO_3_^−^/Br^−^ ratio of 1.4 × 10^−3^ ± 6.6 × 10^−4^. The most superficial samples collected in April 2022 showed higher values of the BrO_3_^−^/Br^−^ ratio, up to 2.2 × 10^−2^ (4.3 nM BrO_3_^−^), while the deepest samples present BrO_3_^−^ concentrations below the instrumental limit of detection (7.8 × 10^−3^ nM; fig. S6), due to rain on snow event on 13 March 2022 (Fig. 2). Regarding Br^−^, as illustrated in fig. S6, no trend is evident within the snowpack, with an average concentration of 160 ± 104 nM.

**Fig. 3.**
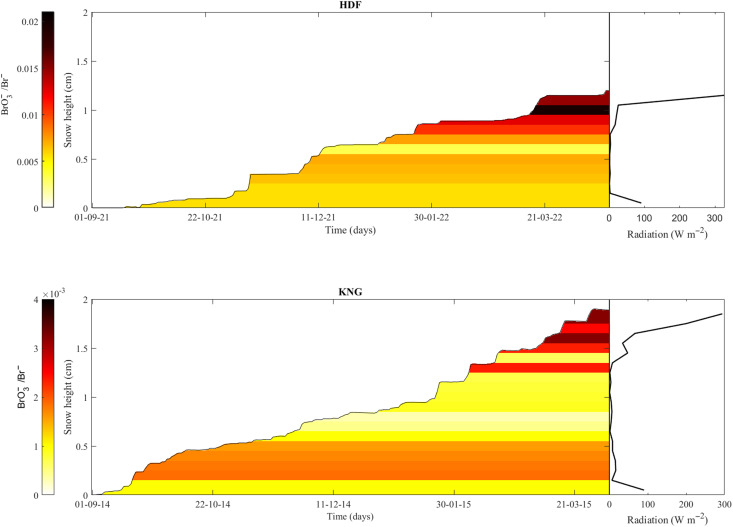
Cumulative BrO_3_^−^/Br^−^ ratio production in glacier snow. Trend of BrO_3_^−^/Br^−^ ratio measurements in seasonal snow samples in the HDF (top, sampled in 2022) and KNG (bottom, sampled in 2015) glaciers. The graphs on the right show the radiation penetrated across 10-cm depth (refer to Materials and Methods), using the incoming radiation data from the CCT. Regarding HDF, sampling took place on 24 April 2022, while regarding KNG, it took place on 10 April 2015.

Last, snow samples from two snow pits, dug in April and in the accumulation area of two glaciers nearby Ny-Ålesund, Holtedahlfonna (HDF) and Kongsvegen (KNG), were analyzed to evaluate the seasonality in bromate production and concentration in the absence of winter melting and percolation events. The results obtained from the 2022 HDF snow pit (Fig. 3) indicate that the BrO_3_^−^/Br^−^ ratio in the lower snow layers is approximately 10 times lower than in the upper layers, ranging from 3.3 × 10^−3^ (bottom) to 4.9 × 10^−2^ observed at the top of the snowpack. Similarly, the 2015 KNG snow pit samples show the highest BrO_3_^−^/Br^−^ ratio near the surface, reaching a maximum of 3.21 × 10^−3^ at a depth of 40 cm. In the middle section of the annual snowpack (90 to 140 cm), the ratio decreases to an average value about four times lower than that in the surface layers, before rising again in the deepest part (140 to 200 cm), where it reaches values comparable to those at the top (see Fig. 3 and Materials and Methods for the site description). At both sites, Br^−^ has an average concentration of 106 ± 60 nM for HDF and 48 ± 41 nM for KNG. As illustrated in fig. S7, in both snow pits, the BrO_3_^−^ appears to increase at the surface, rising from values of ~5.1 × 10^−1^ ± 0.2 × 10^−1^ nM in the deeper samples to a maximum of 2.3 ± 0.5 nM near the surface for HDF. In contrast, for KNG, the lowest layers show values of 9.5 × 10^−2^ ± 4.3 × 10^−2^ nM, while the maximum value of 3.8 × 10^−1^ is found in the layer at a depth of 30 cm.

## DISCUSSION

### The relationship between bromate production and sunlight

The ultimate source of bromine in the snowpack is sea salt aerosols originating from the oceans. Possible mechanisms for BrO_3_^−^ formation in the snowpack include (i) gas-phase reactions during the atmospheric transport of sea salt bromide followed by deposition and (ii) in situ production through oxidation reactions of bromine species in the snowpack.

The first hypothesis is challenged by analyses of aerosol samples collected in Ny-Ålesund from February 2022 to June 2022 (see table S2), which found no traces of BrO_3_^−^ despite showing a trend of methanesulphonic acid (MSA) consistent with prior work in the same area and a Br^−^ concentration ranging from 1.39 to 20.8 ng m^−3^. The absence of BrO_3_^−^ in aerosol samples, despite the presence of Br^−^, suggests that its formation likely occurs after deposition, within the snowpack itself. In our preliminary data analysis, we observed a correlation between BrO_3_^−^ and MSA concentrations (Pearson’s *r* = 0.81, *P* < 0.001; see table S4) in the snowpack, indicating a relationship between the two species. It is well known that the formation of MSA occurs through atmospheric photo-oxidative reactions of dimethylsulfide, a compound released during algal blooms in the atmosphere. Therefore, our first hypothesis is that BrO_3_^−^ may originate from photochemical processes in the atmosphere after being released as bromide from the same marine source as MSA. A second possibility is that the production processes for MSA and BrO_3_^−^ are both linked to solar radiation but in different contexts (i.e., atmosphere versus snowpack), leading to only an apparent correlation between their final concentrations. The connection with photochemistry has been documented for MSA in the atmosphere, but not for bromate in snow.

Considering the daily sampling of surface snow in Ny-Ålesund (Fig. 1), the observed increase, during sunrise, of the BrO_3_^−^/Br^−^ ratio began after a 2-day snowfall followed by a regular sunlight illumination cycle, with bromate concentrations continuously increasing and peaking after ~5 days. The radiation data (Fig. 1B) were collected at the Amundsen-Nobile climate change tower (CCT), located 500 m from the snow sampling site. Radiation reaching the surface is typically extinguished within the first 10 cm of snow (*23*). For samples that were never exposed to sunlight, the BrO_3_^−^/Br^−^ ratio values remained low and relatively constant. However, during sunrise, the BrO_3_^−^/Br^−^ ratio values in the sunlight-exposed layers gradually increased, suggesting an effective mechanism for BrO_3_ production triggered by sunlight (Fig. 2). The formation of the snowpack in the HDF experiment was characterized by a few but intense snowfalls (~15 cm), allowing prolonged exposure of the surface layer to solar radiation (Fig. 3A). In contrast, the KNG snowpack experienced several less intense snowfalls (<10 cm) that reduced the exposure time of the surface snow layer to sunlight (Fig. 3B). Consistently, we observed the lowest BrO_3_^−^ values in the deepest dark snow layers, whereas the illuminated surface layers showed an increase in concentration.

**Fig. 4.**
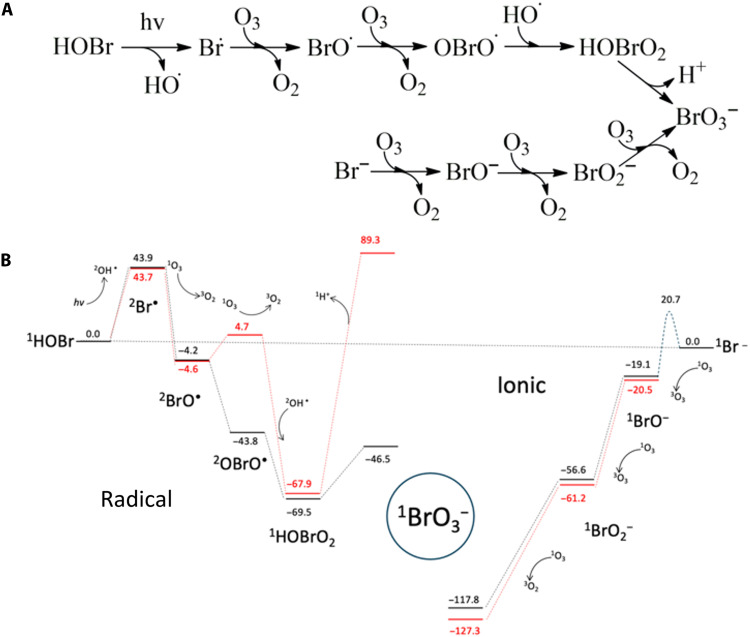
Reaction pathways and potential energy surfaces for the oxidation of Br^−^ to BrO_3_^−^ via radical and ionic mechanisms. (**A**) Reaction pathway and (**B**) potential energy surface of the radical and ionic mechanisms computed at WB97X-D3BJ level of theory. Gas-phase results are reported in red, while calculations performed with the implicit solvent model C-PCM(Water) are reported in black.

The results indicate at least two mechanisms for BrO_3_^−^ formation. The first, related to sunlight intensity, involves the photochemical production of radicals in the snowpack. Specifically, the reaction between BrO and BrONO_2_ generates HOBr in solution, which, under UV irradiation, produces Br radicals, subsequently oxidized by the OH radical (Fig. 4A, top) (*21*). A second mechanism, also active in the dark, may follow an ionic pathway mediated by O_3_ and/or the OH radical, similar to that observed for Cl^−^ in Arctic and Antarctic snow samples (*32*–*34*), as shown in Fig. 4A (bottom) and in the Supplementary Materials. This pathway is well known in wastewater treatment. In this case, starting from Br^−^, a series of oxidative steps leads to the formation of BrO_3_^−^. Laboratory studies have investigated BrO_3_^−^reduction reactions in ice by stimulating specific processes. Since these experiments are conducted in controlled environments, it is plausible that BrO_3_^−^reduction in natural snow is negligible. Both computational and experimental studies support the idea that the oxidation reaction of Br^−^ can occur at the gas-liquid interface (*35*–*38*). It is reasonable to hypothesize that bromate production might happen within the concentrated brine regions that form during freezing. These brines consist of highly concentrated aqueous solutions located between ice grains or at the ice-air interface. It has been demonstrated that within such brines, dissolved species can undergo a substantial increase in concentration, with some studies reporting up to a 10,000-fold enhancement (*24*, *39*).

To shed light on these hypotheses, we conducted a quantum mechanical calculation (see the Supplementary Materials for details) to ensure a consistent understanding of the proposed reaction mechanisms. The photodissociation of HOBr triggers the first mechanism (Fig. 4B, left) and proceeds through radical intermediates. The second mechanism (Fig, 4B, right) involves only ionic species and begins with the oxidation of Br^−^ by ozone. Both mechanisms involve multiple spin transitions, necessitating the calculation of sections of potential energy surfaces with different spin multiplicities. In addition, calculations were performed both in the gas phase and in the presence of water using the implicit solvent model C-PCM (Water).

The potential energy surface of the proposed radical mechanism initiated by photo-absorption is shown in Fig. 4 (left-hand side), which reports the relative free energies of all intermediates computed in the gas phase (red) and in water (black). The photodissociation of HOBr requires an initial energy of 43.7 kcal/mol. Notably, while all the successive steps involving the oxidation of Br are considerably exergonic and irreversible in water, the situation changes markedly in the gas phase. In particular, when simulated in the gas phase rather than in water, one step (the third) even switches from exergonic to endergonic.

The right-hand of Fig. 4 displays the computed section of the potential energy surface for the alternative ionic mechanism. The steps of this mechanism were found to be exergonic in both the presence and absence of the solvent. The first step of the ionic mechanism presents an energy barrier which was further examined by (i) calculating the transition states for both the singlet and triplet states and (ii) identifying the minimum energy crossing point between their potential energy surfaces. Two possible pathways were found. In the “early” crossing scenario (fig. S12), the singlet-triplet crossing occurs before the transition state, while in the “late” crossing case (fig. S13), it occurs afterward. The latter corresponds to the minimum energy path, with an associated energy barrier of 20.7 kcal/mol.

The existence of notable reaction barriers at the entrance of both radical and ionic channels is a possible explanation for the generally low efficiency of bromate formation in the snowpack. However, the initial reaction barrier associated with the photodissociation of HOBr in the radical mechanism can be overcome by the action of sunlight. On the other hand, the ionic mechanism may operate even in the dark but with low efficiency due to the energy barrier associated with crossing singlet-triplet potential energy surfaces.

The calculations suggest that the radical mechanism should occur in the brine layer, while the ionic mechanism could also be effective in the interstitial air of the snowpack. Reactive species such as OH radicals can be formed from the photolysis of NO_3_^−^. Nevertheless, the correlations shown in table S3 do not demonstrate a direct link between BrO_3_^−^ production and NO_3_^−^. This may be due to the consumption of reactive species by other compounds, leading to an absence of a direct correlation with NO_3_^−^, which could produce, in large excess, the reactive species useful for the oxidation of Br^−^. Furthermore, the observed correlation value between BrO_3_^−^ and Br^−^ indicates that bromide is present in excess of the reactive species. This suggests that it should not be considered a limiting reagent and that UV radiation is the primary driver for BrO_3_^−^ formation.

### Implication of bromate formation in snowpack

Our study shows that bromate forms in the Arctic snowpack because sunlight causes bromide oxidation, trapping an important portion of bromine (up to 5%) and reducing the pool of reactive bromine. Although this percentage may seem small, it is particularly relevant from a modeling perspective. Bromate is the most stable form of bromine, meaning that this amount can stay locked in seasonal snow cover, lessening bromine available for bromine explosions. This process, which had not been considered before, could enhance current atmospheric and geochemical models of the bromine cycle.

The snowpack, particularly under solar irradiation, emerges as a highly oxidizing environment, emphasizing the need for further studies on its role in polar region chemistry. Future experimental and theoretical investigations are necessary to refine the kinetic parameters governing bromate formation and stability in snow. The inclusion of a nonreactive bromine pool is a key element in improving the implementation of predictive models of the ozone depletion process, helping to reduce prediction errors, particularly during periods characterized by a marked day-night cycle.

Furthermore, the accumulation of bromate in Arctic snow during winter implies its potential release into the environment during the melting season, which has implications for biogeochemical cycling. Beyond the Arctic, this mechanism may also apply to other snow-covered regions, including Antarctica and mid-latitude mountain ranges like the Alps. Understanding bromate formation in these environments could offer broader insights into the role of snowpack photochemistry in atmospheric halogen cycles and its effects on climate-related processes

## MATERIALS AND METHODS

### Snow pit sampling

Nearby GSRS [78.55°N, 11.53°E; 50 m above sea level (a.s.l.); fig. S1], a weekly snow pit sampling was done to collect snow samples and physical parameters such as temperature and density. Daily surface snow samples were collected between 28th April and 3rd May 2023 at 12 midday. For the seasonal study, three series of samples taken during the polar night and another three series during the all-day period were used.

Snow samples from glaciers were collected from a snow pit near stake 3 (78.95°N, 13.40°E, 600 m a.s.l.) on the HDF ice cap on 24 April 2022. HDF is the largest ice cap (~300 km^2^) on north-western Spitsbergen, about 40 km from the Ny-Ålesund station. It is distributed over an elevation range of 0 to 1241 m a.s.l. The front of KNG is located ~20 km to the east of Ny-Alesund extending about 20 km inland. Samples were collected at stake 8 (78.45°N, 13.20°E, 750 m a.s.l.) on 10 April 2015 (see the Supplementary Materials). No permits or authorizations were required for the collection of the samples used.

After excavation, ~10 cm of snow was removed from the pit wall to create a clean sampling surface and avoid contamination introduced during excavation. The snow wall was sampled using polyethylene precleaned tubes with a depth interval of 10 cm so that 12 samples were taken for each snow pit. Snow pit samples were transported frozen (−20°C) and in dark conditions directly to the Institute of Polar Sciences–National Research Council (ISP-CNR) laboratories in Venice, where they were melted at room temperature under a laminar flow bench (class 100) inside a clean room.

### Aerosol sampling

Aerosol samples were collected using a PM10 high-volume air sampler (TE-6070) operating at a flow rate of 68 m^3^ hour^−1^. The sampler was fitted with an 8″ × 10″ quartz fiber filter that had been precombusted for 4 hours at 400°C in a muffle furnace. Field blanks were obtained by placing the same filters on the sampler with the air pump turned off. Both samples and blanks were stored and transported at −20°C in dark conditions. Bromate was extracted by sonication (30 min) in ultrapure water (10 ml) (*40*).

### Analytical measurements

Snow speciation analyses were carried out by an ion chromatography system (Dionex-2100, Thermo Fisher Scientific) coupled with inductively coupled plasma sector field mass spectrometer (Element XR, Thermo Fisher Scientific). The chromatographic separation was performed on a Dionex Ion AS19 anion exchange column (2 mm by 250 mm) with a Dionex IonPac AG19 guard column (2 mm by 50 mm). The instrumental detection (LOD) calculated were 5 × 10^−2^ nM for Br^−^ and 8 × 10^−3^ nM for BrO_3_^−^, respectively (*41*).

Quantification of BrO_3_^−^ in the aerosol samples was performed with an Anion Exchange Chromatography-Pulsed Amperometric Detection (HPAEC, Dionex, Thermo Fisher Scientific, ICS-5000, Waltham, USA) coupled to a TSQ Altis Plus triple quadrupole mass spectrometer (Thermo Fisher Scientific, ICS-5000, Waltham, USA) using a heated electrospray source operating in negative mode. Chromatographic separation, carried out with a KOH gradient, was performed using a Dionex IonPac AS24 RFIC 2 mm–by–250 mm anion-exchange column (Thermo Fisher Scientific) equipped with a Dionex IonPac AG24 RFIC 2 mm–by–50 mm guard column (Thermo Fisher Scientific) (*40*).

Major anions were determined using an ion chromatography system (Thermo Fisher Scientific, Dionex ICS-5000, Waltham, MA, USA), coupled to a single quadrupole mass spectrometer (MSQ Plus, Thermo Fisher Scientific, Bremen, Germany), while cations were determined using the same ion chromatograph coupled to a conductivity detector, following a previously published method (*42*). A multianion standard for ion chromatography [Cl^−^ (10 mg liter^−1^), NO_3_^−^ (20 mg liter^−1^), Br^−^ (20 mg liter^−1^), and SO_4_^2−^ (20 mg liter^−1^)] and single standard solution of MSA (1000 mg liter^−1^) were purchased from Sigma-Aldrich. The multication standard solution for ion chromatography (Ca^2+^, Na^+^, and NH_4_^+^) at the concentration of 100 mg liter^−1^ was provided by Sigma-Aldrich (*43*). Correlation matrices are available in the Supplementary Materials.

In the case of BrO_3_^−^ values that fell below the LOD, these were not included in the calculation of the BrO_3_^−^/Br^−^ ratio. Furthermore, in the color bar of the graphs, these values were represented in gray.

### Snowpack modeling and snow pit dating

To reconstruct the evolution of the snowpack during the season, a coupled energy balance–snow modeling system was used (*5*, *6*) It simulates multilayer snow density, temperature, and water content with <5-cm vertical resolution, in response to water transport, storage, and refreezing, as well as gravitational compaction and heat diffusion. The model is based on (*5*) but uses a new description of fresh snow density and snow compaction following (*6*). Here, the model is forced with meteorological data from the Norwegian Hindcast Archive (NORA3) reanalysis dataset (*44*), providing fields at 3-km spatial resolution, which are then downscaled to a 1-km resolution grid as described in (*5*). From the model output, we extract seasonal snow height as a function of time for the period from 1 September (2014 for KNG and 2021 for HDF) to the day of sampling. Model values were then rescaled to the snow depth measured on the day of sampling to remove biases while maintaining temporal variability. It is noteworthy that the NORA3 reanalysis dataset assimilated weather station data, e.g., from the Ny-Ålesund weather station, thereby assuring similar meteorological conditions close to weather station locations. The radiation for each 10-cm layer was calculated by integration of the following formula below, considering the 10-cm photic zone

$$R_{d}=R_{0}\text{exp}\left(\frac{d-d_{0}}{z_{e}}\right)$$

where *R_d_* is the radiation at depth d, *R*_0_ is the incident radiation [data acquired at the CCT (*45*)], *d* − *d*_0_ is the depth difference (in this work, assumed 1 cm), and *z_e_* is the e-folding depth, assuming it to be constant (equal to 2 cm^−1^) considering the photic zone to be 10 cm and assuming the snowpack to be constant in terms of microstructure and density.

### Computational details

All calculations were carried out using the ORCA quantum chemistry program package (*46*, *47*). Unless otherwise specified, density functional theory calculations were carried out using the WB97-X exchange correlation functional (*48*). Dispersion corrections were incorporated using the Grimme’s D3 correction combined with the Becke-Johnson damping functions (D3BJ) (*49*). The def2-TZVP basis set was used (*50*). Implicit solvation effects in water were accounted for using the C-PCM method (*51*, *52*). Thermal corrections were computed at 298 K.

## Supplementary Materials

### This PDF file includes:

Supplementary Text Tables S1 to S6 Figs. S1 to S13 References
