# Supplementary material for: Seasonal bromate formation in the Arctic snowpack: Implications for the bromine biogeochemical cycle
Source: Sci Adv. 2026 Apr 17;12(16):eaea3286. doi: 10.1126/sciadv.aea3286 (PMC13089331; doi:10.1126/sciadv.aea3286)
Supplement: Supplementary file 1 — Supplementary Text Tables S1 to S6 Figs. S1 to S13 References [file sciadv.aea3286_sm.pdf]

Science Advances
AAAS

Supplementary Materials for

Seasonal bromate formation in the Arctic snowpack: Implications for the bromine biogeochemical cycle

Stefano Frassati *et al.*

Corresponding author: Stefano Frassati, stefano.frassati@unive.it; Elena Barbaro, elena.barbaro@cnr.it; Andrea Spolaor, andrea.spolaor@cnr.it

Sci. Adv. **12**, eaea3286 (2026)
DOI: 10.1126/sciadv.aea3286

This PDF file includes:

Supplementary Text
Tables S1 to S6
Figs. S1 to S13
References

Supplementary Text

Site description

Gruvebadet Snow Research Site (GSRS), is a clean area dedicate to snow research activities ~1 km southwest from the research village of Ny-Ålesund (78.55°N, 11.53°E, 43 m a.s.l., Figure S 1)(53). Specifically, samples were collected during the polar night on 21 and 28 December 2021 and 4 January 2022, at sunrise on 23 February 2022 and 1 and 9 March 2022, and during the period of maximum sunlight on 3, 11 and 18 May 2022. Density, temperature profile and water content are shown in Figure S 2. On 16 March 2022, a rain-on-snow event shows that liquid water percolates into the snowpack and is also confirmed by the increase in temperature. Furthermore, since that day, the density of the deep layers has remained unchanged at around 450 kg m^{-3}.

The HDF snow pit was dug on April 24, 2022 on the Holthedalfona glacier (78.95° N, 13.40° E) at an elevation of about 600 m a.s.l. Being located on a flat area of the ice field, the sampling site is not significantly affected by shadows due to the surrounding orography. A coupled energy balance-snow modelling system (54, 55) was used to reconstruct the evolution of the snow cover during the considered season. Based on a model simulation between September 2021, corresponding to the beginning of the snow season, and the sampling date in April 2022, there were 6 distinct snowfall event with at least 15 cm of snow accumulation (see Figure S3 for snowpack evolution)

In addition, to assess the potential effect of different snow accumulations rates, which control the snow layer exposure to sunlight, snow samples from a previous snow pit dug on April 10, 2015 were also analyzed. The snowpit was dug in the accumulation area of the KNG glacier at an altitude of 730 m a.s.l. (78.45° N, 13.20° E). The snow samples collected from the snowpit represented the 2014-15 snow season, i.e. from September 2014 to the sampling date. Compared to the 2021-22 snow season recorded in the HDF snow samples, the modelled 2014-2015 snow season at KNG was comparatively different with more frequent snow precipitation events (11 in the whole season) but of lesser intensity with around 7 cm of snow accumulation on average for each snowfall events (see Figure S4 for snowpack evolution)

Bromine and bromate evolution

In this section, we present the concentration of Br^- and BrO_3^- at each site. For all sites, the BrO_3^-trend is continuously increasing from the moment when part of the snowpack is exposed to

light. . As far as Br^- is concerned, only the daily samples (Figure S 5) show a behaviour that tends to increase during the period considered. This is the reason why it was decided to use the Br^-/ BrO_3^- ratio. Figure S 6and Figure S 7 show the concentrations of Br^- and BrO_3^- at 10 cm intervals for HDF and KNG. The concentrations of Br^- are comparable between the two sites, while those of BrO_3^- show significant differences, with values approximately two orders of magnitude lower than those of Br-.

The concentration of Br^- and BrO_3^- in aerosols are shown in Table S2

Statistical analysis

We also determined chloride (Cl^-), nitrate (NO_3^-), sulphate (SO_4^{2-}), sodium (Na^+), ammonium (NH_4^+), calcium (Ca^{2+}), and methanesulphonic acid (MSA) in each sample. The results for the major ions show no trend across all snowpits, while MSA demonstrates increasing trends in the samples corresponding to the spring period, consistent with other studies conducted in the same region(56, 57). In all samples, the concentrations of Cl^- (2.3 ± 1.8 ng g^{-1}) and Na^+ (1.8 ± 1.5 µg g^{-1}) exhibit trends similar to Br^-, with a mean of 10.9 ± 7.7 ng g ^{1}.No trends were observed for SO_4^{2-} and NH_4^+, with average values of 643 ± 553 ng g^{-1} and 53.8 ± 38.5 ng g^{-1}, respectively. Additionally, Ca^{2+} (268 ± 191 ng g^{-1}) and NO_3^- (59.2 ± 47.9 ng g^{-1}) showed no correlations with other ions.

Here we present the Pearson's correlation between the ions presented in this work for all sites Table S1) and for each sampling area (GSRS, HDF and KNG, Table S 2Table S ,and Table **S**4 respectively). With the exception of NO_3^-, Ca^{2+} and BrO_3^-, all ions demonstrate correlations with an average value exceeding 0.7. With regard NO_3^- and Ca^{2+}, no correlations were observed with the other ions analysed in this study.

Conversely, both globally and at specific sites (with the exception of KNG), there is a significant correlation (>0.7, p-value <0.001) between BrO_3^-/Br^- ratioand MSA. This can be attributed to the fact that both can be produced by photo-induced reactions. The former is produced by photo-oxidation in snow, while the latter appears to be a photoproduct of the degradation of DMS in the atmosphere.

Computational validation and results

This computational protocol was validated by means of state-of-the-art DLPNO-CCSD(T) calculations employing the the def2-QZVP basis set and its matching auxiliary correlation counterpart(58-61). TightPNO setting were used in DLPNO-CCSD(T) calculations.

For the sake of comparison, the results obtained with WB97X-D3BJ/def2-TZVP were also compared with those from M062X/def2-TZVP (M062X)(*10*) and B3LYP-D3BJ/def2-TZVP (B3LYP)(62-65).

As mentioned in the main text, two possible mechanisms were plausible, denominated the 'radical mechanism' and the 'ionic mechanism'. Both mechanisms involve multiple spin transitions, requiring the calculation of several sections of potential energy surfaces with different spin multiplicities. For calibrating our computational methodology, we initially focused on the first step of the ionic mechanism in the singlet potential energy surface, which sees bromide and ozone as reactants and BrO^- and oxygen as products. This step was investigated at different levels of theory.

Gas-phase results computed with WB97X-D3BJ were initially compared to the data available in literature at the coupled cluster level(40), revealing good agreement in the ΔG values between the reactant and product complexes. The transition state structure for this step was identified using a combined Nudged Elastic Band (NEB)(66) and transition state optimization approach. Frequency calculations confirmed that the TS presented an imaginary frequency associated with the reaction coordinate. The reaction barrier in the gas phase for the first step was found to be 14.87 kcal mol^{-1}, which is more than 20.0 kcal mol^{-1} lower than the one reported in the literature for the same step(40). The geometry and energetics of the transition state are in good agreement with more recent works(67,68). To verify the dependence of these results by the nature of the exchange-correlation functional, the transition state was optimized with different functionals, specifically M062X and B3LYP-D3BJ. The resulting geometries were consistent across all methods (Figure S 8).

Furthermore, single point calculations were performed at the DLPNO-CCSD(T) level (Figure S 9) on the NEB "images" along the Minimum Energy Path connecting reactants and products. These results indicate that a transition state with the same energy is also present in the DLPNO-CCSD(T) potential energy surface.

These results indicate that WB97X-D3BJ/def2-TZVP is a robust computational methodology for the chemistry investigated in this work, providing results that are consistent with those from

state-of-the-art DPNO-CCSD(T) calculations. The potential energy surface of the proposed radical mechanism initiated by photo-absorption was explored at the WB97X-D3BJ level of theory. Figure S 10 reports the relative free energies of all intermediates computed in the gas-phase (red) and in water (black). Notably, while all the steps involving the oxidation of Br are significantly exergonic and irreversible in water, the situation changes dramatically in the gas phase. In particular, when simulated in the gas phase rather than in water, one step (the third) even switches from exergonic to endergonic. The computed section of potential energy surface for the alternative ionic mechanism is shown in Figure S 11. All steps of this mechanism were found to be exoergonic both in the presence and absence of the solvent.

The first step of the ionic mechanism was further examined by (i) calculating the transition states for both the singlet and triplet states, and (ii) identifying the minimum energy crossing point (MECP) between their potential energy surfaces. Two possible pathways were found. In the "early" crossing scenario (Figure S **12**), the singlet–triplet crossing occurs before the transition state, while in the "late" crossing case (Figure S **13**), it occurs afterward. The latter corresponds to the minimum energy path, with an associated energy barrier of 20.7 kcal mol^{-1}.

In summary, both the radical and ionic mechanisms initially hypothesized were tested computationally to assess their energetic feasibility. Our results on the first step of the ionic mechanism contribute to clarify the mechanistic picture, resolving discrepancies present in previous studies regarding the reaction barrier and transition state energetics. The singlet–triplet minimum energy crossing point (MECP) was also determined, showing that the minimum energy path follows a "late" crossing scenario. Overall, the results demonstrate that both pathways are energetically plausible, with computed energetics consistent with previous high-level studies and experimental observations, thereby offering a more complete and reconciled mechanistic understanding of the reactions.

Rate coefficients

This section reports the rate coefficients relating to the reactions considered in this work, both for the gas phase and for the liquid phase. It is important to emphasise, however, that the system presented represents a simplified version of reality. As regards reactions in water, there are mechanisms that can lead to both ionic and radical processes, with possible interconversions between the two, and their evolution is strongly influenced by the pH of the solution(69). Reactions

between two radical species are expected to be of low probability due to their short lifetimes and low steady-state concentrations; therefore, this pathway is unlikely to represent the dominant mechanism underlying the reported observations.

Furthermore, bromine reactions, particularly in the atmosphere, involve additional reactive species, such as halide radicals, and can lead to the formation of nitrogen-containing brominated compounds.

Table S1 Rate coefficients for the formation of BrO_3^- for both the ionic and radical mechanisms in the liquid phase (T=20 °C) and gas phase. a) refers to the direct photolysis of HOBr and is expressed in s^{-1}(69-71)

	Reaction	Liquid phase (M^{-1} s^{-1})	Gas phase (cm^3 $molecule^{-1}$ s^{-1})
Ionic pathway	1) $Br^- + O_3 \rightarrow BrO^- + O_2$	160	$6.2 \pm 0.4 \times 10^{-15}$
	2) $BrO^- + O_3 \rightarrow BrO_2^- + O_2$	100	$3.9 \pm 0.6 \times 10^{-10}$
	3) $BrO_2^- + O_3 \rightarrow BrO_3^- + O_2$	> 10^5	$1.7 \pm 0.2 \times 10^{-10}$
Radical pathway	4) $HOBr \xrightarrow{h\nu} Br\cdot$	-	3.5×10^{-1} (a)
	5) $Br\cdot + O_3 \rightarrow BrO\cdot + O_2$	1.5×10^9	1.2×10^{-12}
	6) $BrO\cdot + O_3 \rightarrow OBrO\cdot + O_2$	-	< 2×10^{-17}
	7) $OBrO\cdot + \cdot OH \rightarrow HOBrO_2 + H^+$	2×10^9	1.5×10^{-15}

Gas-Phase Geometries

HOBr

H	-4.56339878248338	0.61366377143180	0.00000095354710
O	-4.03479211049220	1.42161356316199	-0.00000296111266
Br	-2.32014710702442	0.82701966540621	-0.00000099243443

OH radical

H	-4.58280756614227	0.61215733239576	0.00000096883511
O	-4.04336643385772	1.42412166760424	-0.00000296883511

O_3 (singlet)

O	-4.72554561160867	0.60728417278327	0.00000000000000
O	-3.71202267729023	1.32006429244264	0.00000000000000
O	-2.60820571110109	0.75684453477409	0.00000000000000

O_2 (triplet)

O	-2.54351144659346	0.78701380504008	0.00000000000000
O	-3.60042355340654	1.34775219495992	0.00000000000000

BrO radical

O	-2.64303662335973	0.70238881717905	0.00000003762152
Br	-0.93598337664027	0.79345518282095	0.00000096237848

$HOBrO_2$

O	-2.00157394693709	0.62909397668552	-0.50188891569318
Br	-0.97162063251158	1.21438476886531	0.57164587162660
O	0.52515031856212	1.01237958676062	0.05920167922934
O	-1.23130223108643	2.97694435205685	0.41395721101071
H	-1.27734450802700	3.14600131563169	-0.54287184617346

OBrO radical

O	-2.59860960240154	0.59439284538030	-0.00000034432939
Br	-0.89821438370138	1.00552873799089	0.00000175560990
O	0.95900298610292	0.59547841662881	0.00000088871948

BrO_3^-

O	-2.03837094773483	0.21365295106821	0.16698901587912
Br	-1.03901960631261	1.34985044937914	-0.46023089655209
O	0.44971702860737	1.06918112606542	0.16244592100892
O	-1.53556347455992	2.79458047348724	0.13085695966405

BrO_2^-

O	-2.07059451319955	0.02968741072565	-0.00001363201284
Br	-1.05105755623712	1.39931825575706	0.00001534472929
O	0.59448006943668	0.94330333351729	-0.00000471271645

BrO^-

O	0.77538741579370	1.06001318309745	-0.00000273664528
Br	-1.01272441579370	1.30326781690255	0.00001273664528

H_3O^+

H	-2.87484328243496	-1.16082305463108	0.56474807270403
O	-2.03302516988351	-1.65047019307125	0.56474824683131
H	-1.18812373544647	-1.16622260999652	0.56474883599050
H	-2.03677581223506	-2.62434114230114	0.56474784447415

H_2O

H	-2.79875032934446	-1.12449582452816	0.56475248126865
O	-2.03317715614540	-1.70555080499124	0.56474330835697
H	-1.27176151451014	-1.11906737048061	0.56475321037438

Reactant Complex (I step ionic mechanism)

O	1.80838077241837	0.63191765072615	-0.06320541139134
O	0.88723296430994	0.85169662704125	0.74094402998243
O	2.44635265433272	-0.47031589886356	0.07679633577350
Br	-1.37134439106103	-0.22585237890385	-0.18075395436459

Product Complex (I step ionic mechanism)

O	2.04012702257491	0.55232452577495	0.20895686041201
O	0.09811130748811	1.04223294433918	0.53004245865055
O	2.09737073116528	-0.58848323895312	-0.28825588697330
Br	-0.75113706122831	-0.39572423116102	-0.03622943208926

WB97X TS singlet (I step ionic mechanism)

O	0.88777230586337	0.45147437741701	-0.23343793890992
O	-0.41715275318584	0.63004264316998	0.56840838848347
O	1.35650573178651	-0.77239277126027	-0.13139706489668
Br	-1.82712528446405	-0.30912424932673	-0.20357338467689

B3LYP TS singlet (I step ionic mechanism)

O	0.96329736417869	0.40641486359530	-0.27169132766212
O	-0.48450225201379	0.59146424268600	0.57825147271478
O	1.52289476063614	-0.74330971807482	-0.11664717878981
Br	-2.00168987280105	-0.25456938820649	-0.18991296626287

M062X TS singlet (I step ionic mechanism)

O	0.89735383204473	0.45499410512733	-0.22063683339984
O	-0.42919065054047	0.64549775508498	0.56388333823004
O	1.32558164000699	-0.77895351944727	-0.14403545669521
Br	-1.79374482151126	-0.32153834076506	-0.19921104813501

Geometries in Water

HOBr

H	-4.56876230798488	0.61358325861446	0.00000095624020
O	-4.03232647345770	1.42019909327604	-0.00000295572981
Br	-2.31724921855741	0.82851464810950	-0.00000100051039

OH radical

H	-4.58335763863539	0.61132936578464	0.00000097285039
O	-4.04281636136460	1.42494963421536	-0.00000297285039

O_3 (singlet)

O	-4.72327027091508	0.60687609681350	0.00000000000000

O	-3.71209000299307	1.32118506515946	0.00000000000000
O	-2.61041372609185	0.75613183802704	0.00000000000000

O_2 (triplet)

O	-2.54371084274094	0.78711959347223	0.00000000000000
O	-3.60022415725905	1.34764640652777	0.00000000000000

BrO radical

O	-2.64111033466249	0.70249157912345	0.00000003866505
Br	-0.93790966533750	0.79335242087655	0.00000096133495

$HOBrO_2$

O	-2.04710425966101	0.32788642191071	-0.15961789151477
Br	-0.95560680654984	1.28798374707466	0.50662132328566
O	0.46806354709357	1.03007439915461	-0.17908184528349
O	-1.48685580487334	2.80520794063390	-0.26023221531015
H	-0.93518767600937	3.52765149122610	0.09235462882276

OBrO radical

O	-2.18253159600738	0.41685032058942	-0.00000085373551
Br	-0.84873813092059	1.34951313613900	0.00000303816389
O	0.49344872692797	0.42903654327158	0.00000011557162

BrO_3^-

O	-2.02938248820914	0.22569258076212	0.17956247332644
Br	-1.03845626254628	1.34741586286595	-0.48812833054845
O	0.43503723917702	1.07250276068417	0.17451606292688
O	-1.53043548842159	2.78165379568776	0.13411079429513

BrO_2^-

O	-2.03614325023371	0.02462801012567	-0.00001393947304

Br	-1.06131864327403	1.42863269476031	0.00001613317048
O	0.57028989350774	0.91904829511402	-0.00000519369743

BrO $^{-}$

O	0.77562048883598	1.05998147585378	-0.00000273866216
Br	-1.01295748883598	1.30329952414622	0.00001273866216

H_3O^+

H	-2.87240801689526	-1.16305679992777	0.56474807307532
O	-2.03309062031246	-1.64989451578252	0.56474824397097
H	-1.19072827207200	-1.16840217658315	0.56474883419383
H	-2.03654109072029	-2.62050350770655	0.56474784875988

H_2O

H	-2.79648621805529	-1.12212338107652	0.56475252098209
O	-2.03316009629504	-1.71027723585735	0.56474323113791
H	-1.27404268564967	-1.11671338306613	0.56475324788000

WB97X TS triplet (I step ionic mechanism)

Br	-1.65210616511160	0.70102708792332	-0.20817886925069
O	0.63849521022778	-1.40576014343890	-0.04818114638024
O	0.87642716674358	-0.24793182789885	0.52384517047018
O	0.13718378814024	0.95266388341443	-0.26748515483925

WB97X TS triplet (I step ionic mechanism)

Br	-1.16433986863109	-0.47947085900669	3.02314938934362
O	0.36737750970475	0.46237725263970	-0.05761120791655
O	-0.61580848351812	0.63520197365425	1.28204753329165
O	0.96153984244446	-0.61802336728726	-0.13428771471872

Early MECP (I step ionic mechanism)

Br	-1.17182374102657	0.53653260602714	-0.14572800180178
O	1.48930884163780	-1.75876017603722	0.07070929561655
O	1.65674287360926	-0.60736788804705	0.61925792884059
O	1.32265702577949	0.42090345805712	-0.17743522265536

Late MECP (I step ionic mechanism)

Br	-0.73334084008927	0.18534127202978	-0.23091965024955
O	1.21318995135563	-1.71688269561053	0.31118195973037
O	1.93206623181912	-0.66845749134983	0.35531050958760
O	0.89471365691451	0.80666491493058	0.02972018093159

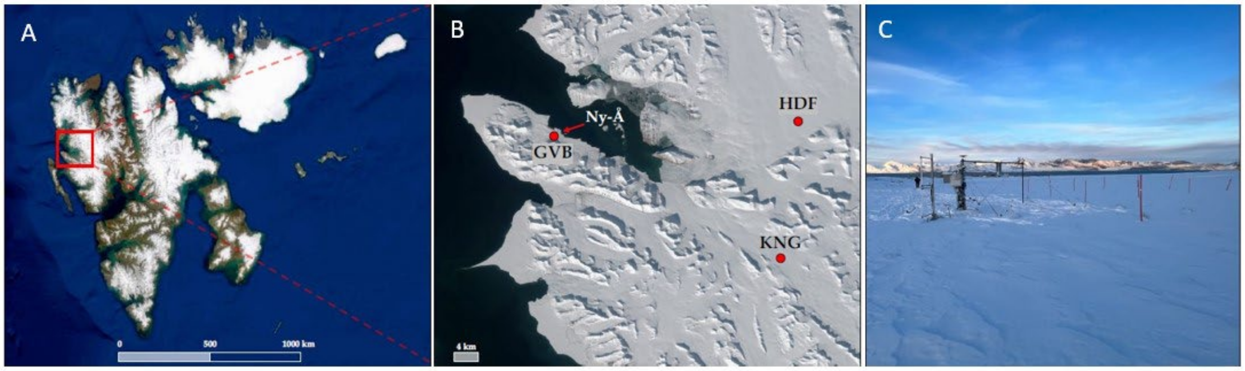

Figure S 1 Sampling sites. Sampling was carried out at the Svalbard archipelago. (**A**)Snowpits were dug in the HDF and KNG glaciers (**B**); seasonal and daily surface snow samples were collected at the GSRS snowfield (**C**).

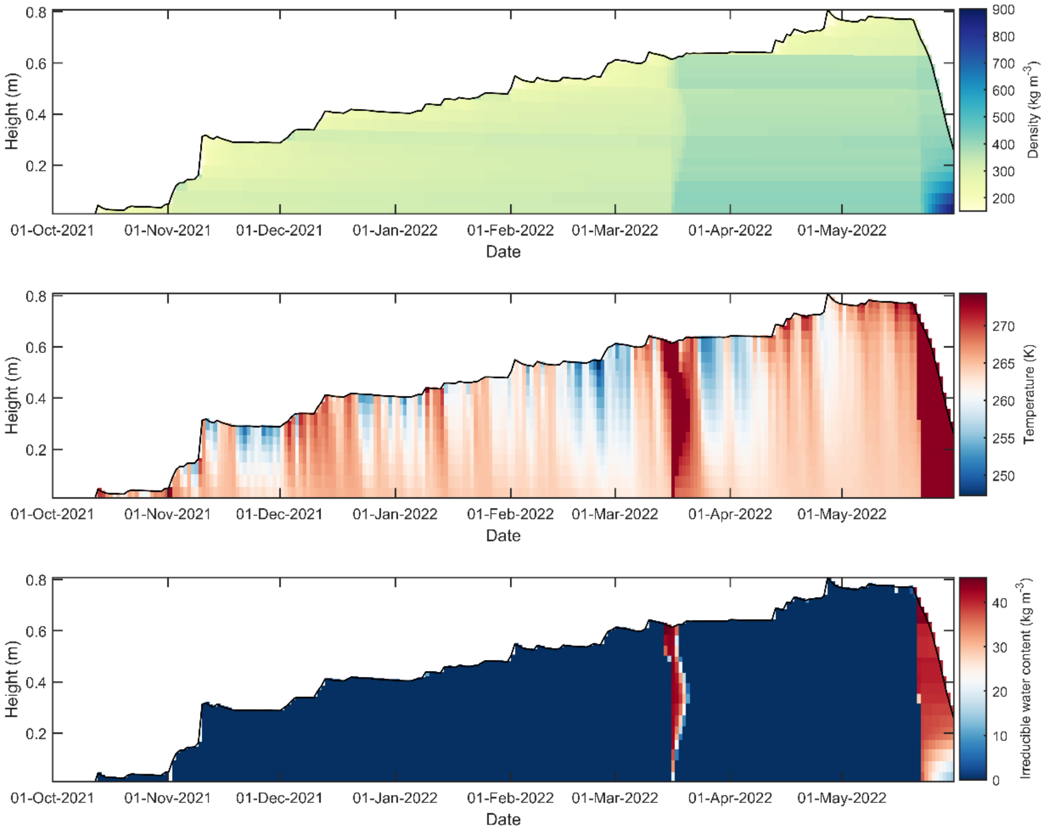

Figure S 2 Model of snowpack evolution at GSRS. Density (top), snow temperature (middle) and water content (bottom).

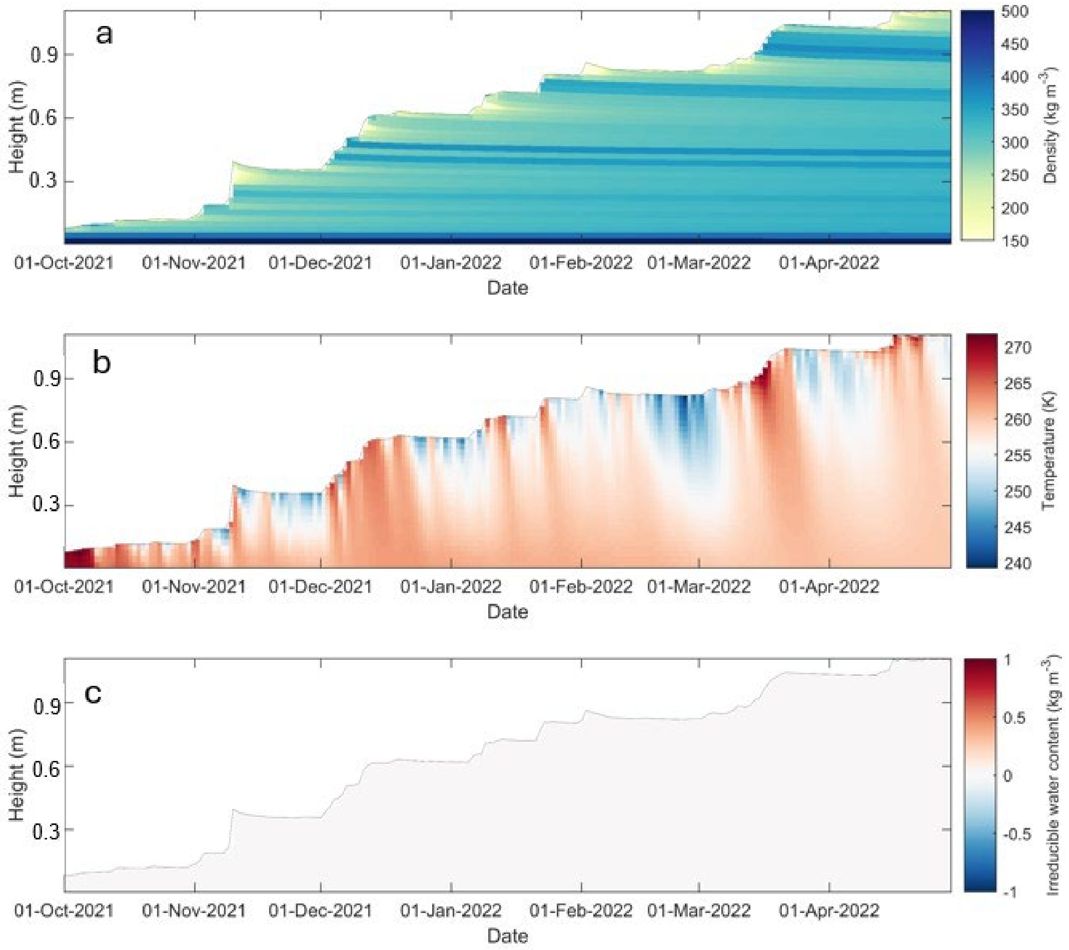

Figure S3 Model of snowpack evolution at HDF. *Density (top), snow temperature (middle) and water content (bottom).*

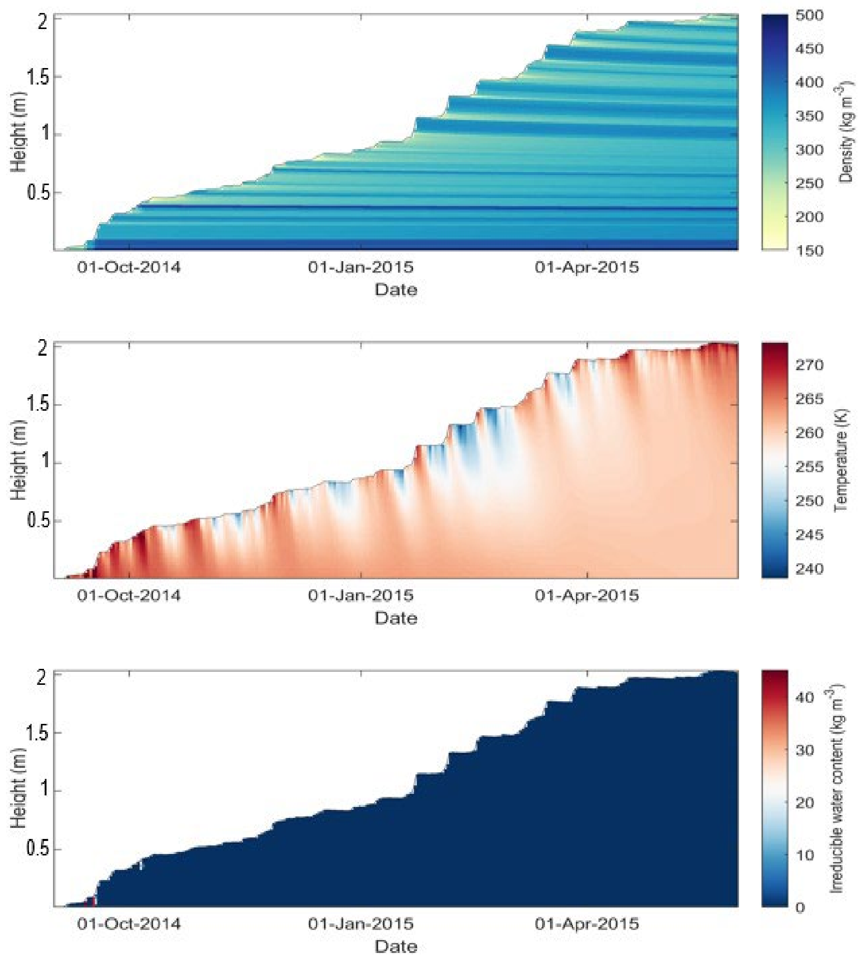

Figure S4 Model of snowpack evolution at KNG. *Density (top), snow temperature (middle) and water content (bottom).*

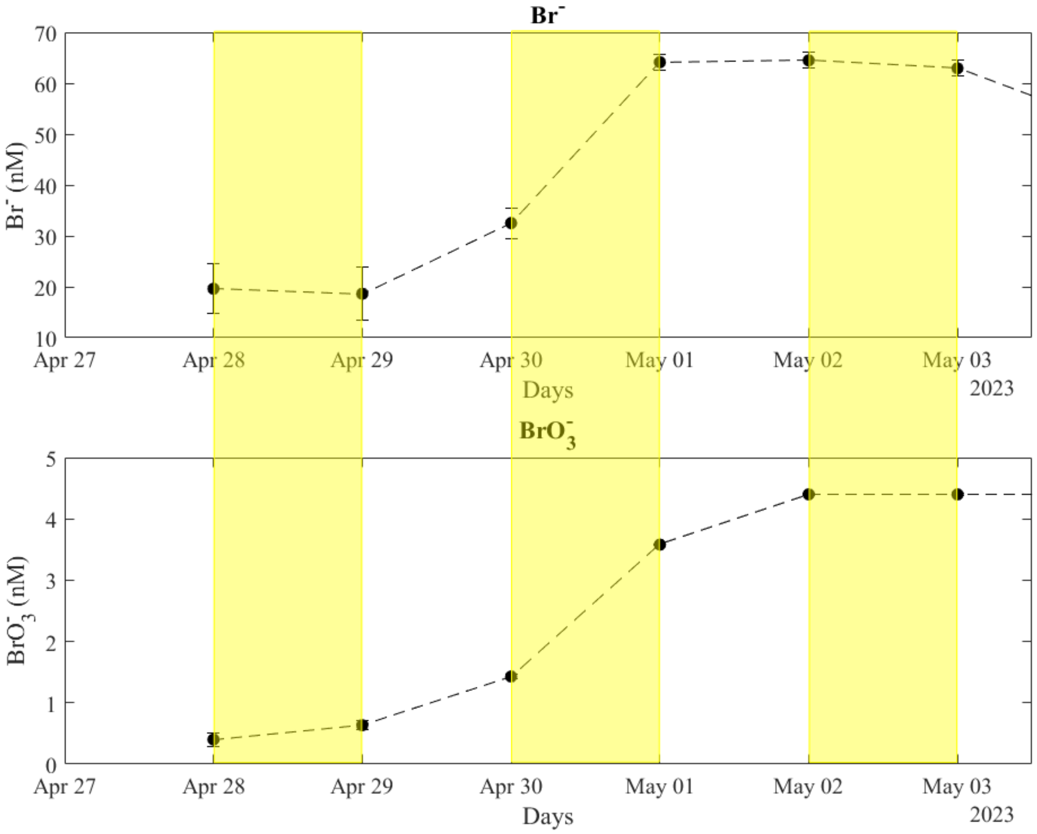

Figure S 5 Bromide (Br^-, top) and bromate (BrO_3^-, bottom) trends during daily surface snow sampling.

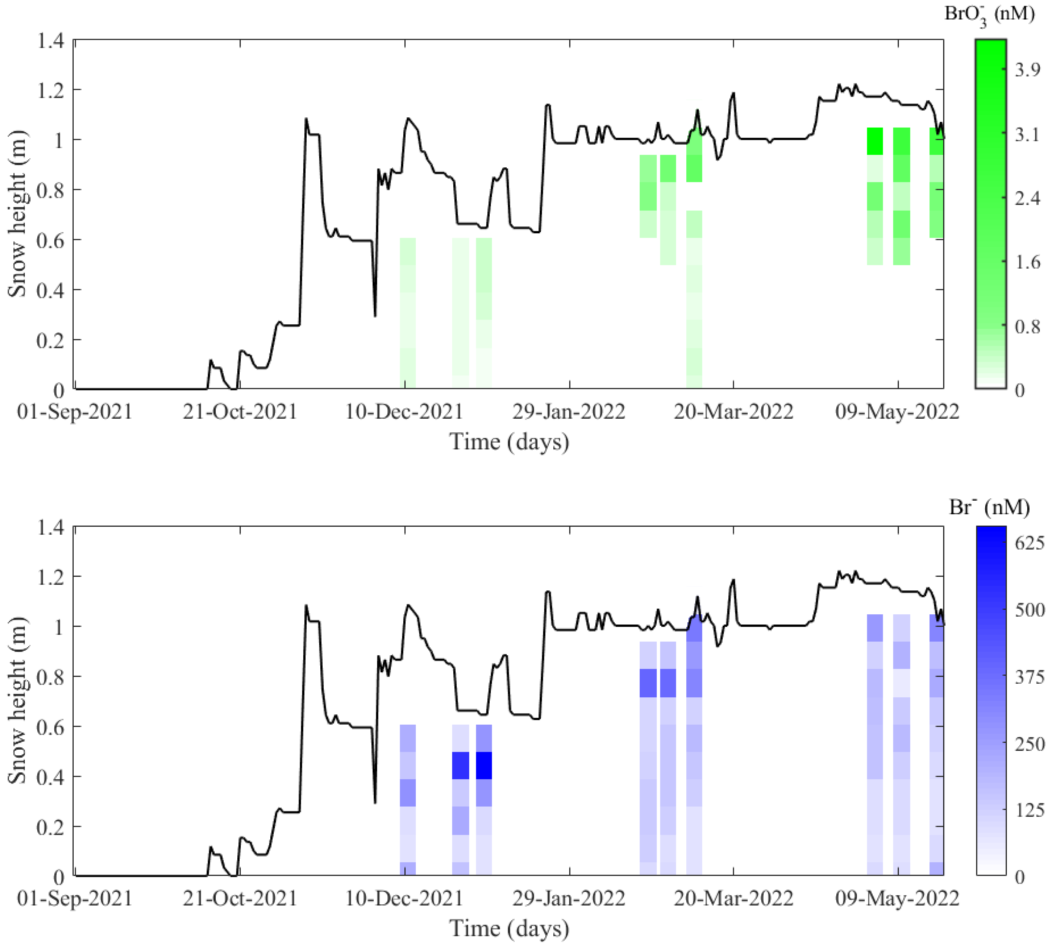

Figure S 6 Annual trend of BrO_3^- top) and Br^-(bottom) in GRSR.

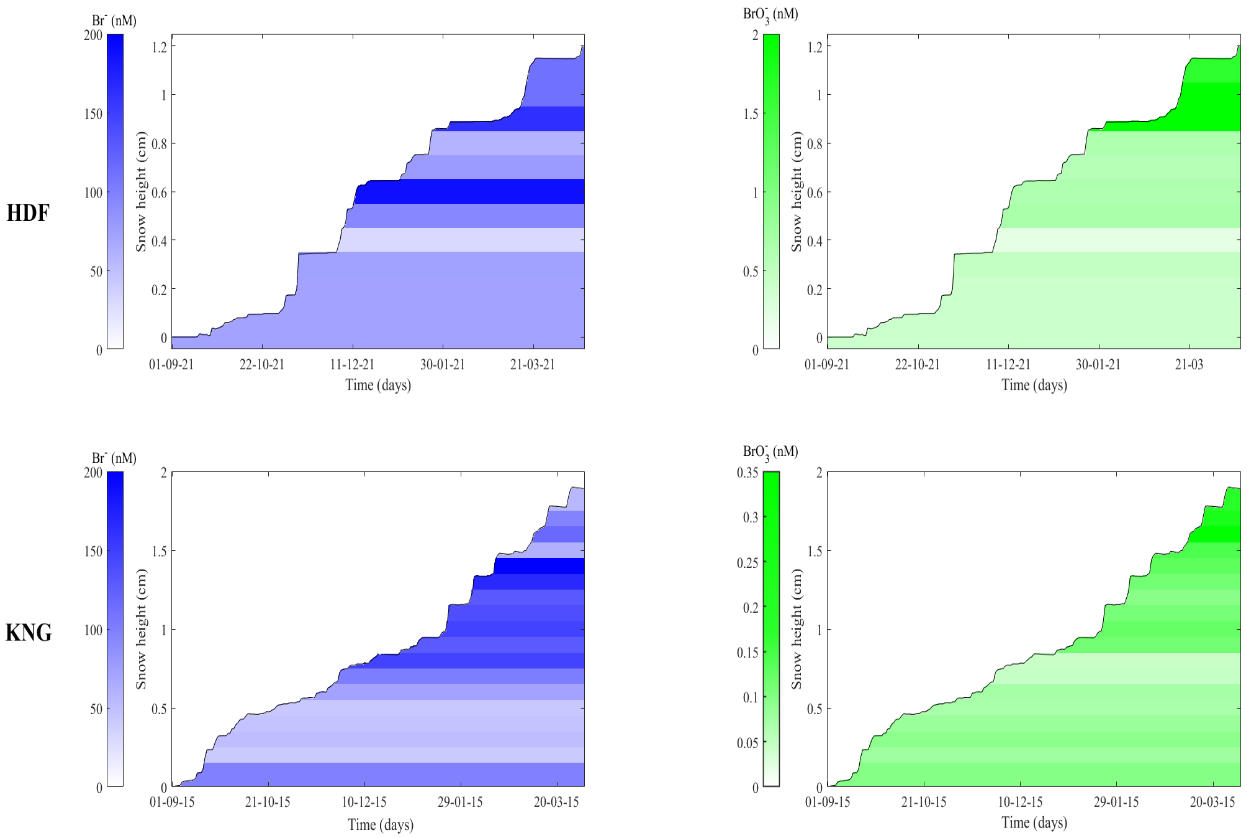

Figure S 7 Annual trend of Br^- (left) and BrO_3^- (right) in HDF (top) and KNG (bottom)

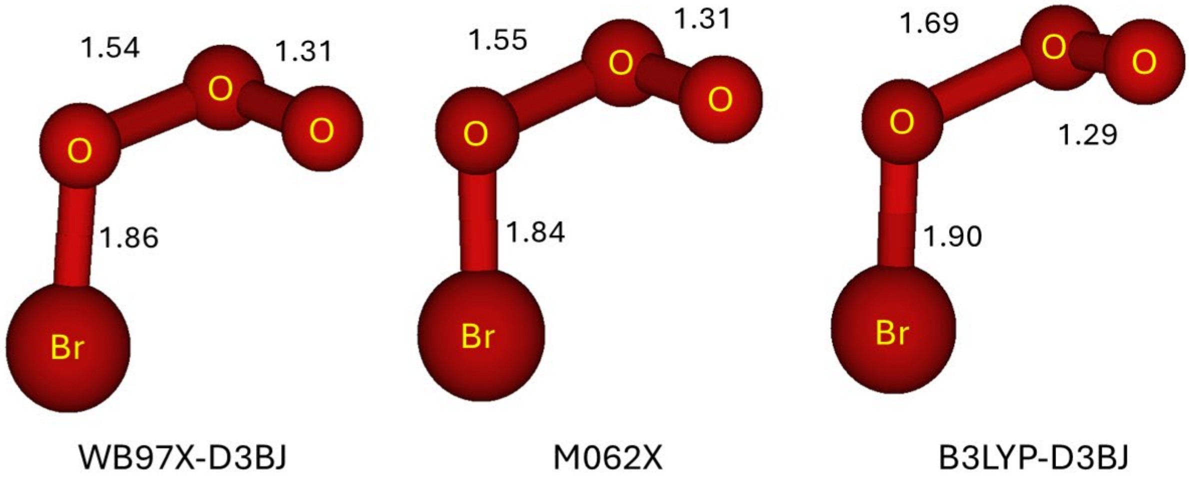

Figure S 8 ***TS structures for the first step of the ionic mechanism.*** *Values were computed at WB97X-D3BJ, M062X and B3LYP-D3BJ levels of theory. Bond lengths are in Angstroms. See text for details.*

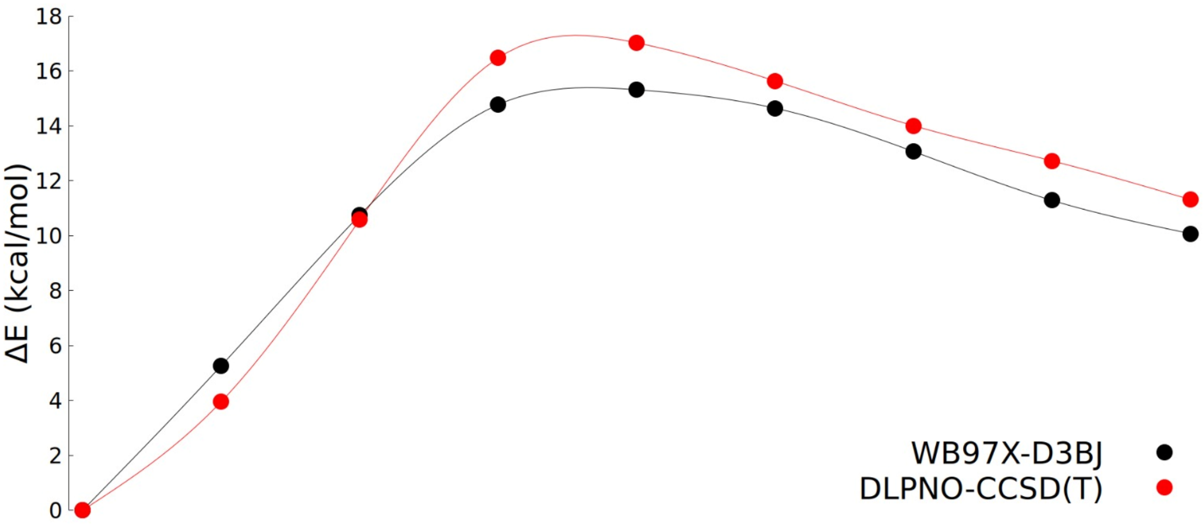

Figure S 9 Section of the singlet potential energy surface connecting reactants and products in the first step of the ionic mechanism. DLPNO-CCSD(T) single-point energy calculations were performed on NEB images optimized at the WB97X-D3BJ level. The computed energies of the NEB images are connected by a solid line as a visual aid along the Minimum Energy Path.

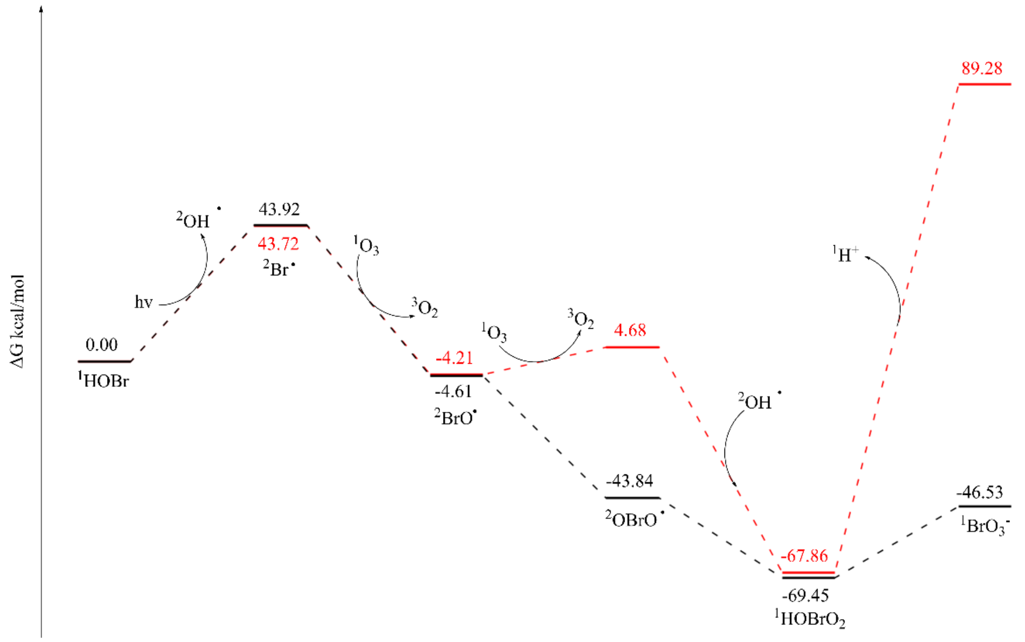

Figure S 10 Potential energy surface of the radical mechanism computed at WB97X-D3BJ level of theory. Gas-phase results are reported in red, while calculations performed with the implicit solvent model C-PCM(Water) are reported in black.

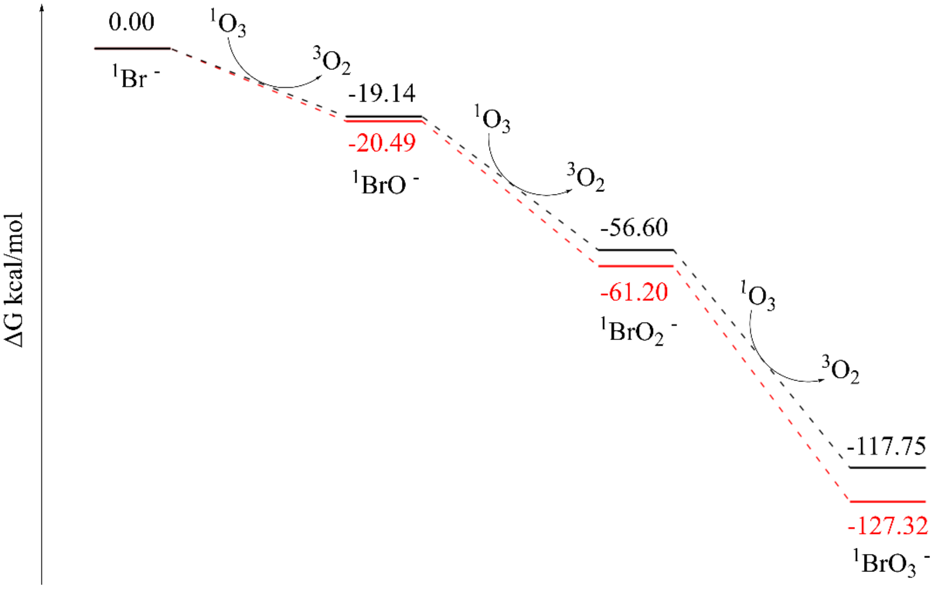

Figure S 11 Potential energy surface of the ionic mechanism computed at WB97X-D3BJ level of theory. Gas-phase results are reported in red, while calculations performed with the implicit solvent model C-PCM(Water) are reported in black.

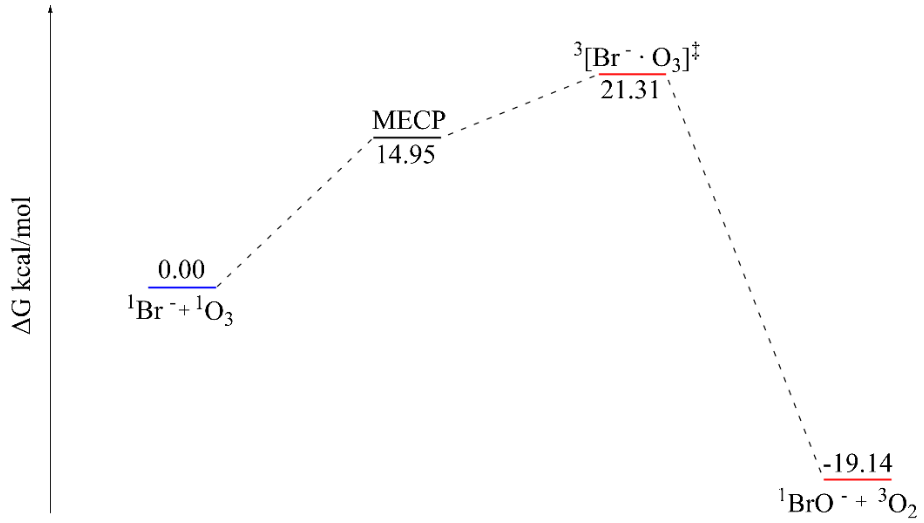

Figure S 12 Early MECP of the first step of the ionic mechanism computed at the WB97X-D3BJ level of theory. Calculations were performed with the implicit solvent model C-PCM(Water). Singlet and triplet states are reported in blue and red, respectively; the MECP is reported in black.

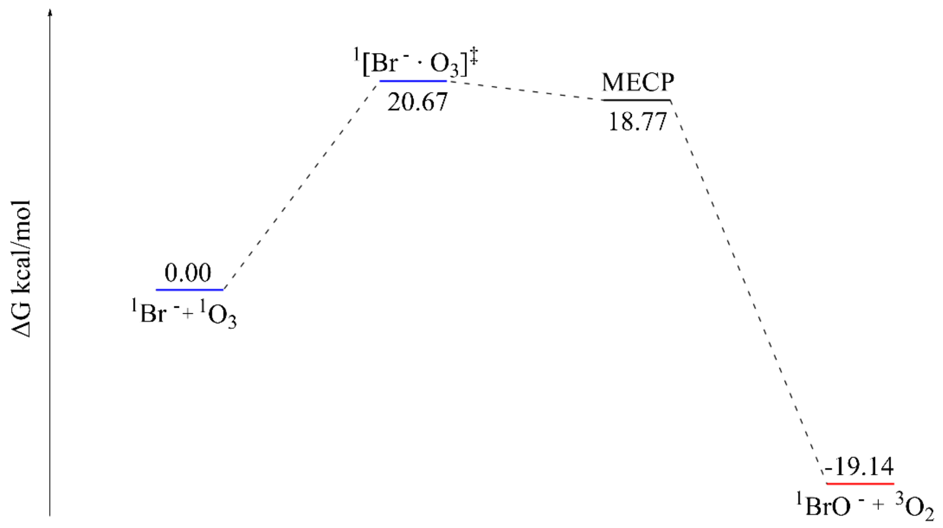

Figure S 13 Late MECP of the first step of the ionic mechanism computed at the WB97X-D3BJ level of theory. Calculations were performed with the implicit solvent model C-PCM(Water). Singlet and triplet states are reported in blue and red, respectively; the MECP is reported in black.

Table S2 Br^-, BrO_3^- data in aerosols at GVB sampled from February 21 to June 8, 2022. MDL for BrO_3^- is $8.34*10^{-4}$ ng m^{-3}

**Start date**	**End date**	**m^3**	**Br^- (ng m^{-3})**	**BrO_3^- (ng m^{-3})**	**BrO_3^-/Br^-**
21/02/2022	28/02/2022	11011	20.82	<MDL	
28/02/2022	07/03/2022	11127	1.69	< MDL	
14/03/2022	21/03/2022	11091	1.45	$6.63*10^{-3}$	$4.58*10^{-3}$
21/03/2022	28/03/2022	10972	4.05	< MDL	
28/03/2022	05/04/2022	12690	4.24	< MDL	
05/04/2022	12/05/2022	11126	4.59	< MDL	
12/04/2022	19/04/2022	11135	2.27	< MDL	
19/04/2022	26/04/2022	11138	2.76	< MDL	
26/04/2022	03/05/2022	11138	3.50	< MDL	
03/05/2022	10/05/2022	11182	7.37	< MDL	
10/05/2022	18/05/2022	12856	3.31	< MDL	
18/05/2022	25/05/2022	11077	2.55	< MDL	
25/05/2022	01/06/2022	11284	3.32	< MDL	
01/06/2022	08/06/2022	11286	1.39	< MDL	

Table S3 Pearson's correlation matrix for ions in all sites. The values in bold indicate a p-value < 0.001.

**All sites**										
	**Br^-**	**BrO_3^-**	**BrO_3^-/Br^-**	**Cl^-**	**NO_3^-**	**SO_4^{2-}**	**MSA**	**Na^+**	**NH_4^+**	**Ca^{2+}**
**Br^-**	1.00									
**BrO_3^-**	0.17	1.00								
**BrO_3^-/Br^-**	-0.08	**0.77**	1.00							
**Cl^-**	**0.95**	0.15	-0.08	1.00						
**NO_3^-**	0.17	0.16	0.18	0.18	1.00					
**SO_4^{2-}**	**0.68**	**0.21**	0.03	**0.76**	**0.34**	1.00				
**MSA**	0.05	**0.66**	**0.72**	0.01	**0.24**	0.17	1.00			
**Na^+**	**0.92**	0.14	-0.08	**0.99**	0.17	**0.76**	0.00	1.00		
**NH_4^+**	**0.73**	**0.43**	**0.23**	**0.73**	0.06	**0.42**	0.08	**0.73**	1.00	
**Ca^{2+}**	**0.27**	-0.07	**-0.20**	**0.35**	0.16	**0.49**	0.10	**0.38**	0.02	1.00

Table S4 Pearson's correlation matrix for ions in GSRS. The values in bold indicate a p-value < 0.001.

**GSRS snow samples**										
	**Br^-**	**BrO_3^-**	**BrO_3^-/Br^-**	**Cl^-**	**NO_3^-**	**SO_4^{2-}**	**MSA**	**Na^+**	**NH_4^+**	**Ca^{2+}**
**Br^-**	1.00									
**BrO_3^-**	0.18	1.00								
**BrO_3^-/Br^-**	0.03	**0.90**	1.00							
**Cl^-**	**0.97**	0.11	-0.02	1.00						
**NO_3^-**	**0.66**	**0.35**	**0.31**	**0.66**	1.00					
**SO_4^{2-}**	**0.91**	**0.33**	0.23	**0.93**	**0.71**	1.00				
**MSA**	0.18	**0.90**	**0.81**	0.10	**0.33**	**0.25**	1.00			
**Na^+**	**0.96**	0.11	-0.02	**1.00**	**0.65**	**0.94**	0.10	1.00		
**NH_4^+**	**0.84**	**0.36**	**0.25**	**0.84**	**0.60**	**0.85**	**0.25**	**0.84**	1.00	
**Ca^{2+}**	**0.38**	0.13	0.04	**0.42**	**0.26**	**0.32**	0.16	**0.42**	**0.36**	1.00

Table S5 Pearson's correlation matrix for ions in HDF. The values in bold indicate a p-value < 0.001.

**HDF snow samples**										
	**Br^-**	**BrO_3^-**	**BrO_3^-/Br^-**	**Cl^-**	**NO_3^-**	**SO_4^{2-}**	**MSA**	**Na^+**	**NH_4^+**	**Ca^{2+}**
**Br^-**	1.00									
**BrO_3^-**	**0.52**	1.00								
**BrO_3^-/Br^-**	-0.18	**0.67**	1.00							
**Cl^-**	**0.95**	**0.55**	-0.14	1.00						
**NO_3^-**	-0.13	-0.02	0.10	-0.33	1.00					
**SO_4^{2-}**	**0.92**	**0.71**	0.09	**0.97**	-0.31	1.00				
**MSA**	-0.19	**0.51**	**0.92**	-0.13	0.07	0.09	1.00			
**Na^+**	**0.95**	**0.56**	-0.14	**1.00**	-0.32	**0.97**	-0.13	1.00		
**NH_4^+**	0.49	0.42	-0.01	**0.53**	-0.47	**0.55**	-0.04	**0.54**	1.00	
**Ca^{2+}**	**0.79**	**0.82**	0.25	**0.87**	-0.29	**0.93**	0.19	**0.87**	**0.58**	1.00

Table S6 Pearson's correlation matrix for ions in KNG. The values in bold indicate a p-value < 0.1.

**KNG snow samples**										
	**Br^-**	**BrO_3^-**	**BrO_3^-/Br^-**	**Cl^-**	**NO_3^-**	**SO_4^{2-}**	**MSA**	**Na^+**	**NH_4^+**	**Ca^{2+}**
**Br^-**	1.00									
**BrO_3^-**	-0.03	1.00								
**BrO_3^-/Br^-**	**-0.55**	**0.76**	1.00							
**Cl^-**	**0.80**	-0.08	**-0.52**	1.00						
**NO_3^-**	0.02	0.03	-0.14	0.25	1.00					
**SO_4^{2-}**	**0.49**	-0.18	**-0.59**	**0.74**	**0.47**	1.00				
**MSA**	-0.32	0.24	**0.42**	-0.17	-0.01	-0.15	1.00			
**Na^+**	**0.74**	0.01	-0.37	**0.96**	0.21	**0.67**	-0.18	1.00		
**NH_4^+**	**0.52**	0.34	-0.04	**0.80**	**0.52**	**0.60**	0.02	**0.82**	1.00	
**Ca^{2+}**	**0.51**	0.04	-0.30	**0.84**	**0.41**	**0.82**	0.09	**0.85**	**0.78**	1.00

REFERENCES

1. W. R. Simpson, R. Von Glasow, K. Riedel, P. Anderson, P. Ariya, J. Bottenheim, J. Burrows, L. J. Carpenter, U. Frieß, M. E. Goodsite, D. Heard, M. Hutterli, H.-W. Jacobi, L. Kaleschke, B. Neff, J. Plane, U. Platt, A. Richter, H. Roscoe, R. Sander, P. Shepson, J. Sodeau, A. Steffen, T. Wagner, E. Wolff, Halogens and their role in polar boundary-layer ozone depletion. *Atmos. Chem. Phys.* **7**, 4375–4418 (2007).

2. A. Saiz-Lopez, R. P. Fernandez, Q. Li, C. A. Cuevas, X. Fu, D. E. Kinnison, S. Tilmes, A. S. Mahajan, J. C. Gómez Martín, F. Iglesias-Suarez, R. Hossaini, J. M. C. Plane, G. Myhre, J.-F. Lamarque, Natural short-lived halogens exert an indirect cooling effect on climate. *Nature* **618**, 967–973 (2023).

3. D. Segato, A. Saiz-Lopez, A. S. Mahajan, F. Wang, J. P. Corella, C. A. Cuevas, T. Erhardt, C. M. Jensen, C. Zeppenfeld, H. A. Kjær, C. Turetta, W. R. L. Cairns, C. Barbante, A. Spolaor, Arctic mercury flux increased through the Last Glacial Termination with a warming climate. *Nat. Geosci.* **16**, 439–445 (2023).

4. K. Abrahamsson, A. Granfors, M. Ahnoff, C. A. Cuevas, A. Saiz-Lopez, Organic bromine compounds produced in sea ice in Antarctic winter. *Nat. Commun.* **9**, 5291 (2018).

5. P. Vallelonga, N. Maffezzoli, A. Saiz-Lopez, F. Scoto, H. A. Kjær, A. Spolaor, Sea-ice reconstructions from bromine and iodine in ice cores. *Quat. Sci. Rev.* **269**, 107133 (2021).

6. K. L. Foster, R. A. Plastridge, J. W. Bottenheim, P. B. Shepson, B. J. Finlayson-Pitts, C. W. Spicer, The role of Br_2 and BrCl in surface ozone destruction at polar sunrise. *Science* **291**, 471–474 (2001).

7. X. Yang, J. A. Pyle, R. A. Cox, N. Theys, M. Van Roozendael, Snow-sourced bromine and its implications for polar tropospheric ozone. *Atmos. Chem. Phys.* **10**, 7763–7773 (2010).

8. K. A. Pratt, K. D. Custard, P. B. Shepson, T. A. Douglas, D. Pöhler, S. General, J. Zielcke, W. R. Simpson, U. Platt, D. J. Tanner, L. Gregory Huey, M. Carlsen, B. H. Stirm, Photochemical production of molecular bromine in Arctic surface snowpacks. *Nat. Geosci.* **6**, 351–356 (2013).

9. T. Tang, J. C. McConnell, Autocatalytic release of bromine from Arctic snow pack during polar sunrise. *Geophys. Res. Lett.* **23**, 2633–2636 (1996).

10. K. D. Custard, A. R. W. Raso, P. B. Shepson, R. M. Staebler, K. A. Pratt, Production and release of molecular bromine and chlorine from the arctic coastal snowpack. *ACS Earth Space Chem.* **1**, 142–151 (2017).

11. F. Burgay, R. P. Fernández, D. Segato, C. Turetta, C. S. Blaszczak-Boxe, R. H. Rhodes, C. Scarchilli, V. Ciardini, C. Barbante, A. Saiz-Lopez, A. Spolaor, 200-Year ice core bromine reconstruction at Dome C (Antarctica): Observational and modelling results. *Cryosphere* **17**, 391–405 (2023).

12. J. E. Dibb, L. D. Ziemba, J. Luxford, P. Beckman, Bromide and other ions in the snow, firn air, and atmospheric boundary layer at Summit during GSHOX. *Atmos. Chem. Phys.* **10**, 9931–9942 (2010).

13. A. Spolaor, E. Barbaro, D. Cappelletti, C. Turetta, M. Mazzola, F. Giardi, M. P. Björkman, F. Lucchetta, F. Dallo, K. A. Pfaffhuber, H. Angot, A. Dommergue, M. Maturilli, A. Saiz-Lopez, C. Barbante, W. R. L. Cairns, Diurnal cycle of iodine, bromine, and mercury concentrations in Svalbard surface snow. *Atmos. Chem. Phys.* **19**, 13325–13339 (2019).

14. L. Marelle, J.-C. Raut, K. S. Law, L. K. Berg, J. D. Fast, R. C. Easter, M. Shrivastava, J. L. Thomas, Improvements to the WRF-Chem 3.5.1 model for quasi-hemispheric simulations of aerosols and ozone in the Arctic. *Geosci. Model Dev.* **10**, 3661–3677 (2017).

15. K. Toyota, J. C. McConnell, R. M. Staebler, A. P. Dastoor, Air–snowpack exchange of bromine, ozone and mercury in the springtime Arctic simulated by the 1-D model PHANTAS – Part 1: In-snow bromine activation and its impact on ozone. *Atmos. Chem. Phys.* **14**, 4101–4133 (2014).

16. S. Falk, B.-M. Sinnhuber, Polar boundary layer bromine explosion and ozone depletion events in the chemistry–climate model EMAC v2.52: Implementation and evaluation of AirSnow algorithm. *Geosci. Model Dev.* **11**, 1115–1131 (2018).

17. S. Zhai, W. Swanson, J. R. McConnell, N. Chellman, T. Opel, M. Sigl, H. Meyer, X. Wang, L. Jaeglé, J. Stutz, J. E. Dibb, K. Fujita, B. Alexander, Implications of snowpack reactive bromine production for arctic ice core bromine preservation. *JGR Atmospheres* **128**, e2023JD039257 (2023).

18. W. F. Swanson, C. D. Holmes, W. R. Simpson, K. Confer, L. Marelle, J. L. Thomas, L. Jaeglé, B. Alexander, S. Zhai, Q. Chen, X. Wang, T. Sherwen, Comparison of model and ground observations finds snowpack and blowing snow aerosols both contribute to Arctic tropospheric reactive bromine. *Atmos. Chem. Phys.* **22**, 14467–14488 (2022).

19. R. P. Fernandez, A. Carmona-Balea, C. A. Cuevas, J. A. Barrera, D. E. Kinnison, J. Lamarque, C. Blaszczak-Boxe, K. Kim, W. Choi, T. Hay, A. Blechschmidt, A. Schönhardt, J. P. Burrows, A. Saiz-Lopez, Modeling the sources and chemistry of polar tropospheric halogens (Cl, Br, and I) using the CAM-chem global chemistry-climate model. *J. Adv .Model Earth Syst.* **11**, 2259–2289 (2019).

20. R. P. Fernandez, L. Berná, O. G. Tomazzeli, A. S. Mahajan, Q. Li, D. E. Kinnison, S. Wang, J.-F. Lamarque, S. Tilmes, H. Skov, C. A. Cuevas, A. Saiz-Lopez, Arctic halogens reduce ozone in the northern mid-latitudes. *Proc. Natl. Acad. Sci. U.S.A.* **121**, e2401975121 (2024).

21. C. M. Morrison, S. Hogard, R. Pearce, A. Mohan, A. N. Pisarenko, E. R. V. Dickenson, U. Von Gunten, E. C. Wert, Critical review on bromate formation during ozonation and control options for its minimization. *Environ. Sci. Technol.* **57**, 18393–18409 (2023).

22. H.-W. Jacobi, B. Hilker, A mechanism for the photochemical transformation of nitrate in snow. *J. Photochem. Photobiol. A Chem.* **185**, 371–382 (2007).

23. A. M. Grannas, A. E. Jones, J. Dibb, M. Ammann, C. Anastasio, H. J. Beine, M. Bergin, J. Bottenheim, C. S. Boxe, G. Carver, G. Chen, J. H. Crawford, F. Dominé, M. M. Frey, M. I. Guzmán, D. E. Heard, D. Helmig, M. R. Hoffmann, R. E. Honrath, L. G. Huey, M. Hutterli, H. W. Jacobi, P. Klán, B. Lefer, J. McConnell, J. Plane, R. Sander, J. Savarino, P. B. Shepson, W. R. Simpson, J. R. Sodeau, R. Von Glasow, R. Weller, E. W. Wolff, T. Zhu, An overview of snow photochemistry: Evidence, mechanisms and impacts. *Atmos. Chem. Phys.* **7**, 4329–4373 (2007).

24. J. P. Bower, C. Anastasio, Measuring a 10,000-fold enhancement of singlet molecular oxygen (1O2*) concentration on illuminated ice relative to the corresponding liquid solution. *Atmos. Environ.* **75**, 188–195 (2013).

25. L. Chu, C. Anastasio, Quantum yields of hydroxyl radical and nitrogen dioxide from the photolysis of nitrate on ice. *J. Phys. Chem. A* **107**, 9594–9602 (2003).

26. C. S. Blaszczak-Boxe, A. Saiz-Lopez, Nitrate photolysis in ice and snow: A critical review of its multiphase chemistry. *Atmos. Environ.* **193**, 224–241 (2018).

27. T. Hullar, T. Tran, C. Anastasio, Nitrate photolysis at the air–ice interface of nature-identical snow. *ACS Earth Space Chem.* **7**, 1791–1797 (2023).

28. J. L. France, M. D. King, J. Lee-Taylor, Hydroxyl (OH) radical production rates in snowpacks from photolysis of hydrogen peroxide (H_2O_2) and nitrate (NO_3^-). *Atmos. Environ.* **41**, 5502–5509 (2007).

29. L. Chu, C. Anastasio, Formation of hydroxyl radical from the photolysis of frozen hydrogen peroxide. *J. Phys. Chem. A* **109**, 6264–6271 (2005).

30. U.S. Environmental Protection Agency, Health Risk Assessment/Characterization of the Drinking Water Disinfection Byproduct Bromate (1998).

31. World Health Organization: Geneva, Bromate in Drinking Water (2003).

32. V. I. Furdui, F. Tomassini, Trends and sources of perchlorate in arctic snow. *Environ. Sci. Technol.* **44**, 588–592 (2010).

33. S. Jiang, T. S. Cox, J. Cole-Dai, K. M. Peterson, G. Shi, Trends of perchlorate in Antarctic snow: Implications for atmospheric production and preservation in snow. *Geophys. Res. Lett.* **43**, 9913–9919 (2016).

34. Y. J. Tham, N. Sarnela, S. Iyer, Q. Li, H. Angot, L. L. J. Quéléver, I. Beck, T. Laurila, L. J. Beck, M. Boyer, J. Carmona-García, A. Borrego-Sánchez, D. Roca-Sanjuán, O. Peräkylä, R. C. Thakur, X.-C. He, Q. Zha, D. Howard, B. Blomquist, S. D. Archer, L. Bariteau, K.

Posman, J. Hueber, D. Helmig, H.-W. Jacobi, H. Junninen, M. Kulmala, A. S. Mahajan, A. Massling, H. Skov, M. Sipilä, J. S. Francisco, J. Schmale, T. Jokinen, A. Saiz-Lopez, Widespread detection of chlorine oxyacids in the Arctic atmosphere. *Nat. Commun.* **14**, 1769 (2023).

35. I. Gladich, J. S. Francisco, R. J. Buszek, M. Vazdar, M. A. Carignano, P. B. Shepson, *Ab initio* study of the reaction of ozone with bromide ion. *J. Phys. Chem. A* **119**, 4482–4488 (2015).

36. Q. Liu, L. M. Schurter, C. E. Muller, S. Aloisio, J. S. Francisco, D. W. Margerum, Kinetics and mechanisms of aqueous ozone reactions with bromide, sulfite, hydrogen sulfite, iodide, and nitrite ions. *Inorg. Chem.* **40**, 4436–4442 (2001).

37. L. Artiglia, J. Edebeli, F. Orlando, S. Chen, M.-T. Lee, P. Corral Arroyo, A. Gilgen, T. Bartels-Rausch, A. Kleibert, M. Vazdar, M. Andres Carignano, J. S. Francisco, P. B. Shepson, I. Gladich, M. Ammann, A surface-stabilized ozonide triggers bromide oxidation at the aqueous solution-vapour interface. *Nat. Commun.* **8**, 700 (2017).

38. S. N. Wren, D. J. Donaldson, J. P. D. Abbatt, Photochemical chlorine and bromine activation from artificial saline snow. *Atmos. Chem. Phys.* **13**, 9789–9800 (2013).

39. C. S. Boxe, A. Saiz-Lopez, Multiphase modeling of nitrate photochemistry in the quasi-liquid layer (QLL): Implications for NO_x release from the Arctic and coastal Antarctic snowpack. *Atmos. Chem. Phys.* **8**, 4855–4864 (2008).

40. M. Feltracco, E. Barbaro, G. Formenton, G. Mazzi, S. Striuli, C. Bragato, R. Piazza, C. Barbante, A. Gambaro, The disinfection by-products are in the air: Aerosol measurements in the urban area of Venice. *Atmos. Environ.* **318**, 120224 (2024).

41. S. Frassati, E. Barbaro, C. Rossetti, G. Cozzi, C. Turetta, F. Scoto, M. Roman, M. Feltracco, K. Kim, C. Barbante, A. Gambaro, A. Spolaor, Inorganic iodine and bromine speciation in Arctic snow at picogram-per-grams levels by IC-ICP-MS. *J. Anal. Sci. Technol.* **15**, 49 (2024).

42. E. Barbaro, E. Morabito, E. Gregoris, M. Feltracco, J. Gabrieli, M. Vardè, W. R. L. Cairns, F. Dallo, F. De Blasi, R. Zangrando, C. Barbante, A. Gambaro, Col margherita observatory: A background site in the Eastern Italian Alps for investigating the chemical composition of atmospheric aerosols. *Atmos. Environ.* **221**, 117071 (2020).

43. E. Barbaro, M. Feltracco, D. Cesari, S. Padoan, R. Zangrando, D. Contini, C. Barbante, A. Gambaro, Characterization of the water soluble fraction in ultrafine, fine, and coarse atmospheric aerosol. *Sci. Total Environ.* **658**, 1423–1439 (2019).

44. I. M. Solbrekke, A. Sorteberg, H. Haakenstad, The 3 km Norwegian reanalysis (NORA3)—A validation of offshore wind resources in the North Sea and the Norwegian Sea. *Wind Energ. Sci.* **6**, 1501–1519 (2021).

45. M. Mazzola, A. P. Viola, C. Lanconelli, V. Vitale, Atmospheric observations at the Amundsen-nobile climate change tower in Ny-Ålesund, Svalbard. *Rend. Fis. Acc. Lincei* **27**, 7–18 (2016).

46. F. Neese, Software update: The ORCA program system—Version 5.0. *WIREs Comput. Mol. Sci.* **12**, e1606 (2022).

47. F. Neese, The ORCA program system. *WIREs Comput. Mol. Sci.* **2**, 73–78 (2012).

48. J.-D. Chai, M. Head-Gordon, Long-range corrected hybrid density functionals with damped atom–atom dispersion corrections. *Phys. Chem. Chem. Phys.* **10**, 6615 (2008).

49. S. Grimme, J. Antony, S. Ehrlich, H. Krieg, A consistent and accurate *ab initio* parametrization of density functional dispersion correction (DFT-D) for the 94 elements H-Pu. *J. Chem. Phys.* **132**, 154104 (2010).

50. F. Weigend, R. Ahlrichs, Balanced basis sets of split valence, triple zeta valence and quadruple zeta valence quality for H to Rn: Design and assessment of accuracy. *Phys. Chem. Chem. Phys.* **7**, 3297–3305 (2005).

51. V. Barone, M. Cossi, Quantum calculation of molecular energies and energy gradients in solution by a conductor solvent model. *J. Phys. Chem. A* **102**, 1995–2001 (1998).

52. M. Cossi, N. Rega, G. Scalmani, V. Barone, Energies, structures, and electronic properties of molecules in solution with the C-PCM solvation model. *J. Comput. Chem.* **24**, 669–681 (2003).

53. W. Van Pelt, V. Pohjola, R. Pettersson, S. Marchenko, J. Kohler, B. Luks, J. O. Hagen, T. V. Schuler, T. Dunse, B. Noël, C. Reijmer, A long-term dataset of climatic mass balance, snow conditions, and runoff in Svalbard (1957–2018). *Cryosphere* **13**, 2259–2280 (2019).

54. L. Van Kampenhout, J. T. M. Lenaerts, W. H. Lipscomb, W. J. Sacks, D. M. Lawrence, A. G. Slater, M. R. Van Den Broeke, Improving the representation of polar snow and firn in the community Earth system model. *J. Adv. Model Earth Syst.* **9**, 2583–2600 (2017).

55. F. Scoto, G. Pappaccogli, M. Mazzola, A. Donateo, R. Salzano, M. Monzali, F. De Blasi, C. Larose, J.-C. Gallet, S. Decesari, A. Spolaor, Automated observation of physical snowpack properties in Ny-Ålesund. *Front. Earth Sci.* **11**, 1123981 (2023).

56. A. Spolaor, C. Varin, X. Pedeli, J. M. Christille, T. Kirchgeorg, F. Giardi, D. Cappelletti, C. Turetta, W. R. L. Cairns, A. Gambaro, A. Bernagozzi, J. C. Gallet, M. P. Björkman, E. Barbaro, Source, timing and dynamics of ionic species mobility in the Svalbard annual snowpack. *Sci. Total Environ.* **751**, 141640 (2021).

57. E. Barbaro, C. Varin, X. Pedeli, J. M. Christille, T. Kirchgeorg, F. Giardi, D. Cappelletti, C. Turetta, A. Gambaro, A. Bernagozzi, J. C. Gallet, M. P. Björkman, A. Spolaor, Dynamics of ionic species in Svalbard annual snow: The effects of rain event and melting. [Preprint] (2019); https://doi.org/10.5194/tc-2019-124.

58. D. G. Liakos, M. Sparta, M. K. Kesharwani, J. M. L. Martin, F. Neese, Exploring the accuracy limits of local pair natural orbital coupled-cluster theory. *J. Chem. Theory Comput.* **11**, 1525–1539 (2015).

59. C. Riplinger, F. Neese, An efficient and near linear scaling pair natural orbital based local coupled cluster method. *J. Chem. Phys.* **138**, 034106 (2013).

60. C. Riplinger, P. Pinski, U. Becker, E. F. Valeev, F. Neese, Sparse maps—A systematic infrastructure for reduced-scaling electronic structure methods. II. Linear scaling domain based pair natural orbital coupled cluster theory. *J. Chem. Phys.* **144**, 024109 (2016).

61. Y. Zhao, D. G. Truhlar, The M06 suite of density functionals for main group thermochemistry, thermochemical kinetics, noncovalent interactions, excited states, and transition elements: Two new functionals and systematic testing of four M06-class functionals and 12 other functionals. *Theor. Chem. Account* **120**, 215–241 (2008).

62. C. Lee, W. Yang, R. G. Parr, Development of the Colle-Salvetti correlation-energy formula into a functional of the electron density. *Phys. Rev. B* **37**, 785–789 (1988).

63. A. D. Becke, A new mixing of Hartree–Fock and local density-functional theories. *J. Chem. Phys.* **98**, 1372–1377 (1993).

64. S. H. Vosko, L. Wilk, M. Nusair, Accurate spin-dependent electron liquid correlation energies for local spin density calculations: A critical analysis. *Can. J. Phys.* **58**, 1200–1211 (1980).

65. P. J. Stephens, F. J. Devlin, C. F. Chabalowski, M. J. Frisch, Ab initio calculation of vibrational absorption and circular dichroism spectra using density functional force fields. *J. Phys. Chem.* **98**, 11623–11627 (1994).

66. G. Henkelman, H. Jónsson, Improved tangent estimate in the nudged elastic band method for finding minimum energy paths and saddle points. *J. Chem. Phys.* **113**, 9978–9985 (2000).

67. D. Trogolo, J. S. Arey, P. R. Tentscher, Gas-phase ozone reactions with a structurally diverse set of molecules: Barrier heights and reaction energies evaluated by coupled cluster and density functional theory calculations. *J. Phys. Chem. A* **123**, 517–536 (2019).

68. S. Brydon, P. Szabó, P. Wongsomboon, B. Poad, D. Marshall, B. Miljevic, A. Trevitt, E. Bieske, J. Harvey, S. Blanksby, Halving the barrier to gas-phase oxidation of bromide by ozone. [Preprint] (2025); https://doi.org/10.26434/chemrxiv-2025-z2rqs.

69. A. Fischbacher, K. Löppenberg, C. Von Sonntag, T. C. Schmidt, A new reaction pathway for bromite to bromate in the ozonation of bromide. *Environ. Sci. Technol.* **49**, 11714–11720 (2015).

70. T. Ingham, D. Bauer, J. Landgraf, J. N. Crowley, Ultraviolet−visible absorption cross sections of gaseous HOBr. *J. Phys. Chem. A* **102**, 3293–3298 (1998).

71. J. B. Burkholder, S. P. Sander, J. Abbatt, J. R. Barker, R. E. Huie, C. E. Kolb, M. J. Kurylo, V. L. Orkin, D. M. Wilmouth, P. H. Wine, Chemical Kinetics and Photochemical Data for Use in Atmospheric Studies: Evaluation No. 19. (JPL Publication 19-5, 2020).
